# Behavioral modifications lead to disparate demographic consequences in two sympatric species

**DOI:** 10.1002/ece3.5472

**Published:** 2019-07-24

**Authors:** Evan P. Tanner, Jeremy P. Orange, Craig A. Davis, R. Dwayne Elmore, Samuel D. Fuhlendorf

**Affiliations:** ^1^ Department of Natural Resource Ecology and Management Oklahoma State University Stillwater OK USA; ^2^ Spatial Epidemiology and Ecology Research Laboratory, Department of Geography University of Florida Gainesville FL USA

**Keywords:** brood, *Callipepla squamata*, *Colinus virginianus*, demography, habitat suitability index, life‐history strategy

## Abstract

Life‐history theory suggests species that typically have a large number of offspring and high adult mortality may make decisions that benefit offspring survival in exchange for increased adult risks. Such behavioral adaptations are essential to understanding how demographic performance is linked to habitat selection during this important life‐history stage. Though studies have illustrated negative fitness consequences to attendant adults or potential fitness benefits to associated offspring because of adaptive habitat selection during brood rearing, equivocal relationships could arise if both aspects of this reproductive trade‐off are not assessed simultaneously. To better understand how adaptive habitat selection during brood rearing influences demographics, we studied the brood survival, attendant parental survival, and space use of two sympatric ground‐nesting bird species, the northern bobwhite (hereafter: “bobwhite”; *Colinus virgininanus*) and scaled quail (*Callipepla squamata*). During the 2013–2014 breeding seasons, we estimated habitat suitability across two grains (2 m and 30 m) for both species and determined how adult space use of these areas influenced individual chick survival and parental risk. We found the proportion of a brood's home range containing highly suitable areas significantly increased bobwhite chick survival (*β* = 0.02, *SE* = 0.006). Additionally, adult weekly survival for bobwhite was greater for individuals not actively brooding offspring (0.9716, *SE* = 0.0054) as compared to brooding adults (0.8928, *SE* = 0.0006). Conversely, brood habitat suitability did not influence scaled quail chick survival during our study, nor did we detect a survival cost for adults that were actively brooding offspring. Our research illustrates the importance of understanding life‐history strategies and how they might influence relationships between adaptive habitat selection and demographic parameters.

## 1 INTRODUCTION

Animal space use is a fundamental pattern in wildlife ecology that constitutes important mechanisms used to conserve and manage species worldwide (Lack, 1933; Manly, McDonald, Thomas, McDonald, & Erickson, 2002; Morrison, Marcot, & Mannan, 2012). However, using this pattern to manage species can be misguided if the assumption that individuals are making selection decisions that maximize their demographic parameters (i.e., survival and/or fecundity) is incomplete. Research has often shown that space use can be misleading indicators of habitat quality (i.e., ecological traps; Bacon et al., 2016; Donovan & Thompson, 2001; Gates & Gysel, 1978; van Horne, 1983) because individuals may not always be able to determine habitat quality and instead rely on environmental cues to guide space use (Kristan, 2003; Storch & Frynta, 1999). Furthermore, this relationship can be highly variable across species and vegetation communities (Bock & Jones, 2004). Thus, determining a link between space use and demographic parameters helps ensure the predictive capabilities of using such patterns for conservation purposes (Beerens, Frederick, Noonburg, & Gawlik, 2015; Folmer & Piersma, 2012).

Further constrained within patterns of habitat selection is an understanding of how adult space‐use patterns influence offspring survival. This is because the patterns of space use for offspring of many species (i.e., broods and neonates) are often ascribed to parental decisions of nest/birth site selection and subsequent habitat use during important life‐history stages for offspring such as the brooding period (Dreitz, 2009; Gibson, Blomberg, Atamian, & Sedinger, 2017; Kolbe & Janzen, 2002; Lengyel, 2006). Such parental decisions play a direct role in influencing offspring survival (Dreitz, 2009; Garrick, Amundson, & Seddon, 2017; Gibson et al., 2017; Kolbe & Janzen, 2002), which in turn can directly affect population dynamics, as this life‐history stage may be a critical period in certain species (Colwell, Hurley, Hall, & Dinsmore, 2007; Sandercock, Jensen, Williams, & Applegate, 2008).

Yet, such behavioral modifications during this important life‐history stage may come at demographic consequences for associated adults (Blomberg, Sedinger, Nonne, & Atamian, 2013; Caudill et al., 2014; Reznick, 1985; Zhao, Fang, Lou, Swenson, & Sun, 2018). Evidence suggests that for species with a large number of offspring and low adult survival (i.e., *r*‐selected species), adult decision‐making should reflect benefits toward offspring survival in exchange for increased adult risks, whereas the opposite tends to be true for species that have longer‐lived adults and fewer offspring (Ghalambor & Martin, 2001). Such increased risks can be associated with physiological constraints (i.e., changes in metabolic requirements during brood rearing [Dawson, Hinsley, Ferns, Bonser, & Eccleston, 2000; Dreitz, 2009]), behavioral changes (i.e., increased vigilance, decreased foraging opportunities, and space‐use change [Williams & Cooke, 1994; Zhao et al., 2018]), and/or changes in biotic interactions such as increased predation risk due to novel behaviors associated with an adult with offspring (i.e., feigning injuries [Bellrose & Holm, 1994; Ghalambor & Martin, 2001]). Thus, adult space use during these periods may be representative of environmental conditions that are more suitable toward offspring survival as opposed to parental survival. These patterns could convolute how models of habitat use are used in conservation planning and wildlife management if demographic consequences of such behavioral modifications are not considered.

Previous research exploring the links between habitat selection during the postnesting period and offspring survival has been inhibited due to logistical constraints and thus has been lacking until recently (Bock & Jones, 2004). Moreover, a dearth of knowledge exists for precocial species, likely due to past logistical constraints associated with highly mobile offspring (Bloom, Clark, Howerter, & Armstrong, 2013; Bock & Jones, 2004; Orange et al., 2016), and the studies that have investigated these species have been somewhat ambiguous in linking habitat selection and offspring survival (Aldridge & Boyce, 2007; Bloom et al., 2013; Dreitz, 2009; Gibson et al., 2017; Gregg & Crawford, 2009; Mathews, Tyre, Taylor, Lusk, & Powell, 2011). Similarly, though many studies illustrate fitness consequences for adults associated with adaptive behavior during the brooding life‐history stage (Blomberg et al., 2013; Hagen, Pitman, Sandercock, Robel, & Applegate, 2011; Mangelinckx, Davis, Allen, Sullivan, & Blomberg, 2018; Zhao et al., 2018), rarely have studies linked adaptive brooding behaviors to offspring survival while simultaneously assessing fitness consequences of the attending adults. By decoupling these two demographic consequences, equivocal results may arise when attempting to link adaptive habitat selection to either offspring survival or attending adult survival separately because this pattern in space use may be more beneficial toward an unobserved demographic parameter rather than the observed parameter (Uboni, Smith, Stahler, & Vucetich, 2017). Thus, attempting to understand concurrent demographic trade‐offs between offspring survival and adult survival associated with brood habitat selection is important when assessing the conservation implications of these behavioral modifications.

**Figure 1:**
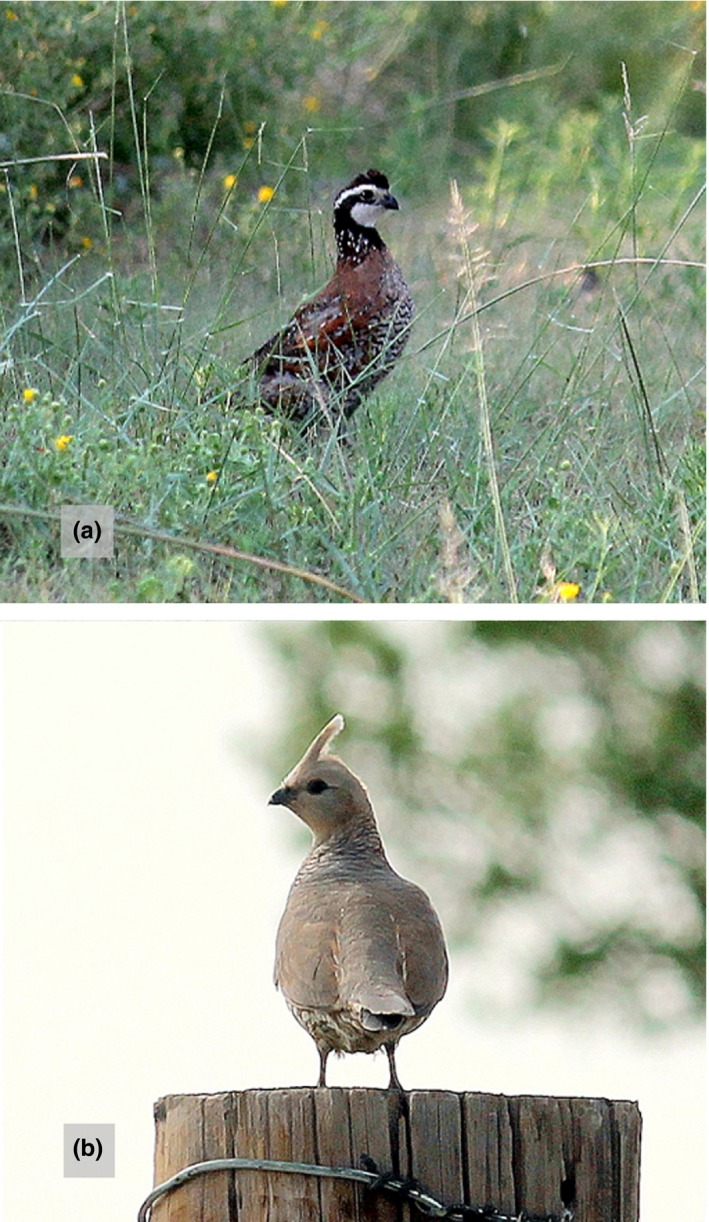
Male northern bobwhite (*Colinus virginianus*; A) and scaled quail (*Callipepla squamata*; B) at Beaver River Wildlife Management Area, Beaver County, Oklahoma, USA

Using radiotelemetry data of individual chicks and brooding adults, we sought to determine whether multiscale habitat suitability indices (HSIs; Bacon et al., 2016; Guisan & Thuiller, 2005) associated with behavioral modifications of brooding adults directly influenced chick survival in two sympatric precocial species of *Galliformes* (northern bobwhite [*Colinus virginianus*; hereafter “bobwhite”] and scaled quail [*Callipepla squamata*]; Figure 1). Furthermore, we sought to determine whether such behavioral modifications by adults resulted in an increased risk for the associated adults by assessing weekly survival probabilities. We chose these two ground‐nesting species as they occur sympatrically in areas of the Great Plains, USA, allowing for a direct comparison on how relative variations in life‐history strategies (Davis et al., 2017) and habitat use during the breeding season (Tanner et al., 2015) influence the link between demographic parameters and behavioral adaptations. The study of sympatric and closely related species may help to better describe the habitat–demographics link and identify factors that may limit population growth and viability of these species (Ackerman, Herzog, Takekawa, & Hartman, 2014; Koons & Rotella, 2003; Sieving, 1992; Varo, 2008). Previous research has indicated divergent reproductive strategies (Davis et al., 2017) within areas of sympatry between these two species (i.e., scaled quail having higher adult survival though generally producing less offspring compared to bobwhite [Rollins, 2000]). Thus, we sought to better understand how these relative differences in reproductive strategies influence how changes in habitat selection are linked to adult and offspring demographic parameters. We hypothesized that (a) greater HSI values would be positively associated with higher offspring survival, that (b) changes in spatial grains across HSIs would result in differing HSI/offspring demography relationships because of the limited mobility that brooding adults have during this life‐history stage, and that (c) behavioral adaptations associated with adults selecting for greater HSI values for offspring survival would result in decreased survival of the attending adults.

## 2 MATERIALS AND METHODS

### 2.1 Study area

We conducted our study at the 11,315‐ha Beaver River Wildlife Management Area (BRWMA) in western Oklahoma (lat 36°50′21.62″N, long 100°42′15.93″W), managed by the Oklahoma Department of Wildlife Conservation. The BRWMA primarily consists of upland rangeland characteristic of a sand sagebrush (*Artemisia filifolia*) community with the floodplain of the Beaver River transecting it. The primary soil types composing BRWMA are Tivoli fine sand soils in the uplands and Lesho silty clay loam in the floodplain.

During our study period (summers of 2013–2014), annual precipitation ranged from 394 mm in 2014 to 503 mm in 2013, with both years having drier conditions than the long‐term (1981–2010) average of 560 mm for this area (Brock et al.., 1995; McPherson et al., 2007). Furthermore, average summer (May–July) temperatures ranged from 25.7 to 27.2°C in 2013 and 25.3 to 30.1°C in 2014, generally exceeding the long‐term average summer temperature of 25.3°C for this region (Brock et al., 1995; McPherson et al., 2007). The study area was under meteorological drought conditions throughout the entirety of our study, with 2013 being under D2 (severe), D3 (extreme), and D4 (exceptional) drought conditions 30%, 40%, and 30% of the year. This is compared to 2014, which was under D2, D3, and D4 drought conditions 24%, 61%, and 15% of the year (The National Drought Mitigation Center [Lincoln, Nebraska, USA], U.S. Department of Agriculture, National Oceanic and Atmospheric Administration).

### 2.2 Bird capture and monitoring

Bobwhite and scaled quail adults were captured using baited walk‐in funnel traps and were fitted with 7‐g necklace‐style VHF radio‐transmitters (Advanced Telemetry Solutions, Isanti, MN). Nests were located via radiotelemetry and monitored daily after they were initially found. After hatching, broods were monitored daily via the radio‐marked adult until the chicks reached 8–12 days old when they were captured for attachment of radio‐transmitters. We used a combination of methods (Andes et al., 2012; Smith et al., 2003) to capture chicks. Following capture, chicks were held in a small portable cooler with a warm water bottle to prevent hypothermia. We attached transmitters to 50%–66% of chicks captured in each brood. Chicks were fitted with 0.45‐g suture‐style transmitters that had an expected battery life of 21–23 days (American Wildlife Enterprises, Monticello, FL). Transmitters were attached using methods described by Burkepile, Conelly, Stanley, and Reese (2002) and Dreitz, Baeten, Davis, and Riordan (2011). Attachment and capture protocols were approved by Oklahoma State University's Institutional Animal Care and Use Committee (ACUP #AG132 and #AG11‐22). We acknowledge that a limitation of our study is that some individual chicks likely experienced mortality before our 8‐ to 12‐day‐old capture period, and thus, all survival estimates and space‐use relationships exclude this initial period of high posthatch mortality (Terhune, Palmer, & Wellendorf, 2019).

Following capture, chicks were located daily. We located broods by homing (White & Garrott, 1990) to a distance of 15–20 m to minimize disturbance. If all radio‐tagged chicks in a brood died, radio‐tagged brooding adults were flushed on a weekly basis to verify the presence of at least one chick with a brooding adult to estimate habitat use and movement. To minimize the effect of variation in diurnal habitat selection that can influence habitat analysis (Taylor, Church, & Rusch, 1999; Taylor & Guthery, 1994), we alternated collection of daily telemetry locations between two time periods: active (sunrise–11:00 and 16:01–sunset) and loafing (11:01–16:00). Daily brood locations were used to calculate a relative index of average daily movement for broods (Tanner et al., 2016). We estimated this index by using the Euclidean distance between daily consecutive brood locations (Brøseth & Pedersen, 2010; Lohr, Collins, Williams, & Castelli, 2011) and averaged them across the population. The average daily movement values for both bobwhite (179.1 m, standard error [*SE*] = 9.6 m) and scaled quail (214.7 m, *SE* = 8.4 m) were used in subsequent analyses described in the following sections.

### 2.3 Habitat suitability analysis for broods

To determine habitat suitability on our study site, we used a maximum entropy algorithm, Maxent version 3.3.3 (Phillips & Dudík, 2008). Traditionally, this algorithm has been used for species distribution models or ecological niche models (Elith et al., 2011). However, Maxent may also be useful in determining smaller scale patterns of space use or habitat selection (Baasch, Tyre, Millspaugh, Hygnstrom, & Vercauteren, 2010; Tanner, Elmore, et al., 2017). We integrated the radiotelemetry locations from our broods as the occurrence dataset for our modeling exercise. Any identical occurrence locations (i.e., multiple locations within the same pixel of our environmental layers) were removed from the dataset prior to running models. Furthermore, we eliminated any broods with occurrence locations or home ranges located outside the extent of our study area as determined by the extent of our environmental layers used for modeling.

#### 2.3.1 Environmental layers

Similar to Tanner, Elmore, et al. (2017) and Tanner, Papeş, et al. (2017), environmental layers used for our Maxent modeling procedure represented the configuration and structure of vegetation within our study site. An initial vegetation layer was created using an Iso Cluster Unsupervised Classification method from 2‐m resolution satellite imagery which was collected in July of 2013. This exercise resulted in a vegetation layer consisting of 10 primary cover types: mixed shrub, sand sagebrush, mixed grass, shortgrass/yucca, sparse vegetation, bare ground, salt cedar, open water, developed areas, and agriculture/food plots. Descriptions of these 10 primary cover types are given in Appendix S1.

To incorporate variability in resource selection across multiple scales, we used environmental layers at both 2 m and 30 m grains (discussed here) and across two extents: the buffered home range and study site extents (discussed in the next section). We incorporated both changes in grain and changes in extent to meet the qualifications of a multiscale study as described by McGarigal, Wan, Zeller, Timm, and Cushman (2016). To incorporate a change in grain, we used Block Statistics and the Resample tools in ArcGIS 10.2 (ESRI, 2011) with a majority rule for classification.

Following the creation of our vegetation layer, we calculated a normalized difference vegetation index (NDVI). Furthermore, we used FRAGSTATS 4.2.1.603 (McGarigal, Cushman, & Ene, 2012) and the vegetation layer to create class‐ and landscape‐level metrics to incorporate as environmental layers for Maxent models. FRAGSTATS metrics were calculated using a round moving window with a radius of 215 m, which was equivalent to the maximum average daily movement of broods across both species during our study. To limit any chance of correlation or redundancy in metrics included in our analysis (Ritters et al., 1995), we selected FRAGSTATS metrics from our study area that were previously shown to be influential in space‐use analyses for bobwhite during the breeding season (Tanner, Elmore, et al., 2017; Tanner, Papeş, et al., 2017). We also excluded any metrics that were highly sensitive to change when incorporating a change in grain size (Lustig, Stouffer, Roigé, & Worner, 2015). Based on these criteria, we included 14 metrics: the coefficient of variation in patch size for mixed shrub, total landscape edge density (m/ha), edge density of specific vegetation types (mixed shrub, sand sagebrush, salt cedar, bare ground, and shortgrass/yucca), mean area of all vegetation patches (m^2^), mean area bare ground and sand sagebrush patches (m^2^), perimeter–area fractal dimension (i.e., shape complexity across all patches), perimeter–area fractal dimension of mixed shrub and sand sagebrush patches, and the contagion index. The contagion index is a measure of interspersion of patch types and the overall patch dispersion, such that it is based on the probability of finding a pixel of type *i* adjacent to a pixel of type *j* (O'Neill et al., 1988). Thus, a value of 0 represents a landscape where every pixel is a different patch type and is maximally interspersed, and a value of 100 represents a landscape where all patch types are maximally aggregated (McGarigal et al., 2012). For all layers, we reclassified “no data” cells within the extent of our study site to 0 before incorporating them into the Maxent algorithm (Foley, Rueda, Peterson, & Wilkerson, 2008).

We also included distance‐based variables that included the Euclidean distance (m) to anthropogenic features including oil/gas structures, artificial surface water sources, and four different types of roads (county road, primary WMA road, restricted access WMA road [truck and all‐terrain vehicle {ATV} access], and restricted access WMA road [ATV traffic only]). We separated roads into four categories to represent varying levels of potential human disturbance (i.e., road traffic), as differences in bobwhite hazard rates have been indicated based on classified road types on our study site (Tanner et al., 2016). We only included functioning artificial water sources (windmills with water tanks, gallinaceous guzzlers, and solar water wells that were providing surface water) in our analysis as they have been shown to influence bobwhite and scaled quail space use on our study site (Tanner et al., 2015). We did not differentiate between functioning and nonfunctioning oil/gas wells to create our distance raster as only 6% of these wells were considered nonfunctioning (Tanner et al., 2016). Spatial oil/gas well data were obtained from the Oklahoma Corporation Commission in 2013, county road data were obtained from the Oklahoma Department of Transportation (http://okmaps.org/ogi/search.aspx), and all other road data were mapped via ground‐truthing efforts. The vegetation, NDVI, distance‐based, and FRAGSTATS layers were included as environmental layers in our initial modeling exercise described in the next section.

#### 2.3.2 Second‐order Maxent modeling

We used a two‐stage approach to model habitat suitability for both bobwhite and scaled quail through Maxent. These two stages included an analysis for second‐order selection (i.e., individual home ranges) and an analysis for the entire population across our study site. Because we had repeated sampling of individuals (radiotelemetry) which were used for the occurrence (or presence) dataset for Maxent models, we initially built Maxent models for individual broods within their buffered home ranges (Baldwin, 2009). Brood home ranges were calculated using a 95% fixed‐kernel method with least squares cross‐validation within the Geospatial Modelling Environment 0.7.2.1 (Beyer, 2012) and were buffered by the species' specific average daily movement patterns (Peters et al., 2015). We estimated home ranges for broods with ≥30 radiotelemetry locations (Seaman & Powell, 1996). To do this, all environmental layers were clipped to an individual's buffered home range. Similar to a backward stepwise variable selection approach (Gherghel, Brischoux, & Papeş, 2018; Hastie, Tibshirani, & Friedman, 2001), we initially eliminated highly correlated variables (|*r|* ≥ 0.7; Dormann et al., 2013) and variables that had ≤5% contribution to model accuracy gain (Phillips, Anderson, & Schapire, 2006; Phillips & Dudík, 2008; Sahlean, Gherghel, Papeş, Strugariu, & Zamfirescu, 2014) for individual brood models. If two variables were highly correlated, we retained the variable that had the highest contribution to model accuracy gain. This resulted in idiosyncratic variable suites for each brood which were derived from our initial 22 environmental variables described above.

Individual Maxent models were initially run using default input values. This included the use of 10,000 background pseudo‐absence points, a regularization multiplier of one, 500 iterations per model, and a convergence threshold of 0.00001. However, if an individual's buffered home range had <10,000 cells, we adjusted the number of background points to represent 90% of the cells within that home range. Models were replicated 100 times using a bootstrap method (Araújo, Marcondes‐Machado, & Costa, 2014), and 25% of occurrence locations were held out as a test dataset to test the validity of our models (Sahlean et al., 2014). We used 10 percentile training presence as the threshold method to estimate the test omission error because this threshold method generally outperforms other threshold rules (Liu, White, & Newell, 2013).

Once all individual brood models were run, we used a Kruskal–Wallis test (Zar, 1999) to determine which variables were most influential in determining habitat suitability across all individuals by species (Baldwin, 2009). Variable influence was measured by each of their contributions to model accuracy gain, and the variables with the statistically greatest contributions to model accuracy gain (based on the results of the Kruskal–Wallis test) were retained. The variables that were retained after this step were then carried forward to build a first‐order Maxent model to determine habitat suitability at the study site extent. All procedures described in this section were completed at both 2 m and 30 m grains.

#### 2.3.3 First‐order Maxent modeling

A first‐order Maxent model was created for both species at both 2 m and 30 m grains using the radiotelemetry occurrence data and the variables that were retained from the steps described in the previous section. All procedures described in the previous section were used to create first‐order Maxent models. However, to account for model complexity, we calibrated our individual models by using different values for the regularization multiplier, which has been shown to significantly influence model performance (Radosavljevic & Anderson, 2014; Tanner, Papeş, et al., 2017). We compared average test omission errors across first‐order models using regularization multiplier values of 0.25, 0.50, 1.00, 1.50, 2.00, 4.00, 6.00, 8.00, and 10.00 (Radosavljevic & Anderson, 2014). Models with different regularization multiplier values were repeated 10 times. We then tested for differences in the average test omission errors of these models through a one‐way analysis of variance (ANOVA) and a post hoc Tukey HSD pairwise comparisons test (*α* = 0.05) using PROC GLM in SAS 9.4. The regularization multiplier value that resulted in the statistically lowest average test omission error was used for the final model for each species across both grains.

We used the logistic output from Maxent to obtain values of habitat suitability for each species across both grains. This ultimately created a map within the extent of our study site in which each cell had a probability of habitat suitability for each species. These values in theory could range from 0 to 1, with 1 representing a cell with 100% probability of habitat suitability for a specific species. These suitability values were then carried forward into our survival analyses to determine how habitat suitability influenced individual chick survival (described below).

### 2.4 Habitat suitability analysis for nonbrooding adults

To determine whether brooding adults were differentially selecting habitat as a function of behavioral adaptations associated with brooding activities, we compared the habitat suitability indices created for brooding adults to habitat suitability indices created for nonbrooding adults using occurrence locations (i.e., radiotelemetry locations) of nonbrooding adults that were obtained during the brooding season for bobwhite and scaled quail (June 23–October 20 and June 9–October 12, 2013–2014, respectively). To create habitat suitability indices for nonbrooding adults, we used the Maxent algorithm and followed an identical protocol as described in the previous three sections. Thus, we obtained two unique nonbrooding habitat suitability indices for each species (i.e., 2 m and 30 m grains) and considered these nonbrooding indices to be representative of what was available to brooding adults based on what the rest of the population was using.

Once pairwise (brooding and nonbrooding) habitat suitability indices for each species at each grain were created, we estimated the amount of similarity between the indices to determine the measure of divergent space use associated with behavioral adaptations of parents with broods. To estimate similarity between indices, we estimated the relative rank (RR) metric (Warren & Seifert, 2011) through ENMTools (version 1.4.4; Warren, Glor, & Turelli, 2010). This metric is an estimate of the probability that any two patches of habitat have the same relative ranking for pairwise models (rather than quantifying similarity based on suitability values) and was used (as opposed to other similarity metrics for niche modeling) because we were interested in the relative prioritization of habitat across pairwise indices (Warren, Wright, Seifert, & Shaffer, 2014). Values range from 0 (low similarity of relative ranking) to 1 (complete similarity of relative ranking). We created a null distribution of the RR metric in ENMTools using the background similarity test function with 100 Maxent pseudoreplications that used a random sample of background pixels as occurrences instead of brooding and nonbrooding locations. We then determined whether the empirical RR values estimated from the pairwise brooding/nonbrooding index comparisons were contained within the null distribution of the RR metrics. If the empirical RR values were not within the null distribution, we considered the RR metric to be statistically significant (thus indicating differential space use between brooding and nonbrooding adults).

### 2.5 Chick survival analysis

To estimate the daily survival probabilities of individual chicks by species, we used the nest survival model in Program MARK (version 9.0; Cooch & White, 2017; White & Burnham, 1999) as it allows for potential gaps in monitoring events and staggered entry of individuals into a population (Dinsmore, White, & Knopf, 2002). As the battery life of our transmitters was ~21–23 days, we estimated daily survival up to 20 days after transmitter attachment. Since chicks were caught ~8–12 days after hatch, this 20‐day period after transmitter attachment was used to represent the stage of a chick's life history in which they are incapable of thermogenesis (Lusk, Guthery, Cox, DeMaso, & Peoples, 2005) and highly reliant on brooding adults for survival.

We attributed mortalities or signal losses that occurred within three days of transmitter attachment to be caused by transmitter failure or capture‐related mortality. If this occurred, the associated chicks were censored from survival analysis (Larson, Clark, & Winterstein, 2001). When radio‐tagged chicks were located at a distance >100 m from the brooding adult, we checked individual chicks for potential mortalities. If fates of chicks were unknown due to loss of transmitter signals, we right‐censored individual history encounter files to the last date they were known to be alive.

Independence of sample units is an important assumption when implementing survival analyses (Cooch & White, 2017) as a violation of this assumption could underestimate sample variances (Schmutz, Ward, Sedinger, & Rexstad, 1995). To test for independence of individual chicks within broods, we first used a bootstrapping simulation process in Program MARK to estimate an overdispersion factor (*ĉ*; Bishop, White, & Lukacs, 2008). A *ĉ* > 1 would be indicative of overdispersion and a lack of independence for individuals within groups, while a *ĉ* < 1 would indicate underdispersion with individuals being highly independent within groups (Bishop et al., 2008). We initially built models that were representative of time trends. This included the covariates linear time, quadratic time, and ordinal date of hatch. Global, additive, and univariate models were built using these three covariates, and the most parsimonious model was considered the model with the lowest Akaike's information criterion (AIC_c_) value adjusted for small sample sizes (Burnham & Anderson, 2002). We then ran a bootstrapping simulation process on the most parsimonious model with 5,000 iterations (Chernick, 1999) and used a unique brood identification number to identify blocks of data. The *ĉ* values obtained from these bootstrap simulations were then applied to the remaining model‐building strategies employed for the entire dataset.

Additionally, we included two variable suites representing daily weather conditions and landscape/vegetation composition (i.e., HSIs). Though our initial hypotheses were related to understanding how adaptive habitat selection of brooding adults influenced offspring survival, we tested effects of weather conditions because they have been shown to significantly influence habitat selection and survival of these species during brood rearing (Carroll, Davis, Elmore, Fuhlendorf, & Thacker, 2015; Terhune et al., 2019). We obtained daily weather conditions from three weather stations (WeatherHawk 232) that were oriented west‐to‐east across our study site (mean distance between weather stations: 13 km). To do this, we used the weather station closest to the centroid of a brood's home range. Conditions were recorded every hour at each weather station. Weather variables included in our analysis were as follows: average daily ambient temperature (°C), maximum daily ambient temperature (°C), minimum daily ambient temperature (°C), average daily variance in ambient temperature, maximum daily solar radiation (W/m^2^), average daily wind speed (km/hr), daily precipitation (mm), and average daily relative humidity (%). We conducted a principal component analysis (PCA) using singular value decomposition in Program R (v.3.4.3) to reduce the amount of variables and correlation among variables within our survival analysis. To determine the optimum number of principal components to retain in our analysis, we used the broken‐stick stopping rule (Jackson, 1993). Based on this stopping rule, we retained two principal components (PCs) for our survival analysis. PC1 (44.7% of explained variance) represented hot dry days, while PC2 (18.8% of explained variance) represented cooler and more humid days with precipitation events (Appendix S2).

Finally, landscape/vegetation composition variables included six variables representing habitat suitability values derived from our final first‐order Maxent models. To derive habitat suitability variables for our survival analysis, we used the logistic probability of suitability maps from our final first‐order Maxent models and separated cells into categories using Jenks natural breaks classification (Jenks, 1967). We chose to limit the number of categories that habitat suitability values are grouped in by selecting the category in which the goodness of variance fit begins to increase at a decreasing rate (i.e., the point of inflection). This was to prevent an arbitrary selection for the number of categories we split our data into. For both species, across both grains, the point of inflection for goodness of variance fit values was three categories (Appendix S3). We arbitrarily refer to these categories as “low,” “medium,” and “high” habitat suitability values (Table 1). Once cells were classified, we calculated the percentage of each brood's home range that was composed of low, medium, and high habitat suitability cells and incorporated these values into our survival analysis.

**Table 1:** Low, medium, and high habitat suitability categories in which cells from rasters derived from population‐level Maxent models were classified based on their individual values. Occurrence data for Maxent models represented radio‐transmitter locations for northern bobwhite (*Colinus virginianus*) and scaled quail (*Callipepla squamata*) broods during the 2013–2014 breeding seasons at Beaver River WMA, Oklahoma, USA. Three categories were chosen based on Jenks natural breaks classification. Percentages represent the percent of our study site classified in the associated category

Species	Grain (m)	Low	%	Medium	%	High	%
Northern bobwhite	2	0–0.150	56	0.151–0.408	24	0.409–0.801	20
	30	0–0.120	69	0.121–0.382	21	0.383–0.844	10
Scaled quail	2	0–0.189	39	0.190–0.430	31	0.431–0.800	30
	30	0–0.192	47	0.193–0.446	32	0.447–0.800	21

We conducted a correlation analysis on all variables and eliminated any variables that were highly correlated (|*r*| ≥ 0.70) before our model‐building exercise. The percentage of medium habitat suitability cells within a brood's home range were dropped for both species and both grains as these variables were highly correlated with other habitat suitability values. We employed a purposeful model‐building strategy (Hosmer, David, Lemeshow, & Sturdivant, 2013) to identify the best performing model for chick survival by species, in which each species was modeled under a different framework. We initially assessed univariate models and retained variables with a *p* < 0.25 to build a global model. We then individually removed variables with a *p* > 0.05 from the global model based on the strength of the *p* value until a model contained only variables that had *p* < 0.05. Variables that were initially eliminated from our analysis were then added back to the reduced global model to determine whether the significance of the relationship had changed after incorporating other additive effects.

Once model building was complete, we determined the most parsimonious model based on the quasi‐AIC_c_ values (QAIC_c_; Burnham & Anderson, 2002). We considered models with a ΔQAIC_c_ < 2 to be competitive models for explaining variability in chick survival and considered variables that had *β* estimates whose 95% confidence intervals excluded 0 to be significant to chick survival (Burnham & Anderson, 2002). Daily survival rates were obtained from the most parsimonious model, and we used the Delta method (Powell, 2007) to compute 20‐day survival rates and associated error rates.

### 2.6 Post hoc parental survival analysis

We estimated daily survival probabilities of radio‐collared bobwhite and scaled quail adults from June 23–October 20 and June 9–October 12, respectively. These two periods corresponded with the same temporal extent of the brood survival analyses associated with this study. To estimate survival probabilities, we use the nest survival model in Program MARK (White & Burnham, 1999). Our objective was to determine whether individuals that were involved with brooding activities were at greater risk of mortality than those that were not brooding. As such, we included three variables in the adult survival analysis: time, quadratic time, and the brooding status of the individual (i.e., either actively brooding or not brooding). Both male and female adults were included in our analysis as both sexes participate in brooding activities. If an individual ceased brooding activities, we right‐censored their encounter history on the last day they were observed brooding and then created a new encounter history beginning on the next day they were observed without a brood (Mangelinckx et al., 2018). We implemented the same purposeful model‐building strategy used in our chick survival analysis and compared explanatory models to a null model. Similarly, we considered models with a ΔAIC_c_ < 2 to be competitive models for explaining variability in adult survival and considered variables that had *β* estimates whose 95% confidence intervals excluded 0 to be significant to adult survival (Burnham & Anderson, 2002).

## 3 RESULTS

During the breeding seasons of 2013–2014, a total of 102 bobwhite (2013 = 45, 2014 = 57) and 95 scaled quail (2013 = 23, 2014 = 72) chicks were captured and fitted with radio‐transmitters. This represented a total of 25 (2013 = 12, 2014 = 13) and 20 (2013 = 6, 2014 = 14) different broods for bobwhite and scaled quail, respectively, in which home ranges and average daily movement patterns were estimated. Though scaled quail broods had larger home ranges compared to bobwhite (scaled quail = 65.84 ha, *SE* = 11.69; bobwhite = 45.58 ha, *SE* = 5.41), they were not statistically different (*t* = −1.57, *p* = 0.13). However, average daily movements of scaled quail broods (214.70 m, *SE* = 8.38, *n* = 429) were greater than bobwhite broods (179.07 m, *SE* = 6.92, *n* = 670; *t* = 3.33, *p* < 0.01).

### 3.1 Habitat suitability modeling for broods

There were no differences in the model performance of regularization multiplier values for both the 2 m (*p* = 0.14) and 30 m (*p* = 0.06) bobwhite brood first‐order models. Therefore, a regularization multiplier value of one was used for both of these models. However, a regularization multiplier value of 10 was used for both the 2 m (*p* = 0.02) and 30 m (*p* = 0.02) scaled quail brood first‐order models as these values were shown to have the best average test omission errors. Based on average test omission errors, first‐order models performed well for both bobwhite (2 m = 0.11, 30 m = 0.14) and scaled quail (2 m = 0.10, 30 m = 0.10) broods.

Habitat suitability analyses at the first‐order resulted in idiosyncratic variable suites for each brooding species across both grains (Table 2; Appendices S4–S7). The 30 m bobwhite brood model and the 2 m scaled quail brood model had >50% of the variability explained by a single variable. Specifically, the distance from county roads explained 72% of the variability for the 30 m bobwhite brood first‐order model and the distance from surface water explained 53.2% of the variability for the 2 m scaled quail brood model. The relationship between HSI and distance from county roads was unimodal for the 30 m bobwhite brood model, in which probability of habitat suitability increased as distance from county roads increased up to approximately 2,000 m (Appendix S5). For the 2 m scaled quail brood model, probability of habitat suitability decreased as the distance from surface water increased up to approximately 5,000 m (Appendix S6). The top contributing variable for the 2 m bobwhite brood model (contagion index; 43.9% variable contribution) indicated a unimodal relationship between habitat suitability and the contagion value, in which the highest probability of habitat suitability occurred at intermediate levels of vegetation interspersion (contagion indices from 41–47). The 30 m scaled quail brood model had the most variable uncertainty based on variable contributions (Table 2). However, the top contributing variable (distance from walk‐in only roads) suggested that probability of habitat suitability decreased as distance from walk‐in only roads increased (Appendix S7). Based on Jenks natural breaks classification, areas that were given high HSI values had the lowest coverage across our study area for both species and across both grains (Table 1).

**Table 2:** Variables used in the final first‐order Maxent models for northern bobwhite (*Colinus virginianus*) and scaled quail (*Callipepla squamata*) broods across two grains during the breeding seasons from 2013 to 2014 at Beaver River WMA, Oklahoma, USA

Species	Grain (m)	Variable	Contribution (%)^a^
Northern bobwhite	2	Contagion index	43.9
		Distance from primary WMA roads (m)	33.3
		Distance from surface water (m)	16.7
		Distance from oil/gas pad (m)	6.1
	30	Distance from county roads (m)	72
		Edge density (m/ha)	12.2
		Distance from ATV only roads (m)	9.2
		Distance from primary WMA roads (m)	6.6
Scaled quail	2	Distance from surface water (m)	53.2
		Coefficient of variation in mixed shrub patch size	25.6
		Distance from county roads (m)	12.1
		Distance from ATV only roads (m)	6.4
		Contagion index	2.7
	30	Distance from walk‐in only roads (m)	29.5
		Coefficient of variation in mixed shrub patch size	26.7
		Distance from surface water (m)	17
		Distance from ATV only roads (m)	11
		Distance from county roads (m)	10.4
		Edge density (m/ha)	5.4

a Contribution represents the average variable contribution to model accuracy gain for MAXENT models.

### 3.2 Comparison of habitat suitability indices for brooding and nonbrooding adults

Occurrence locations from 76 bobwhite and 42 scaled quail nonbrooding adults were used to create first‐order habitat suitability indices for nonbrooding individuals. Average variable contribution to model accuracy gain and idiosyncratic relationships with probability of habitat suitability and environmental covariates for nonbrooding models can be found in Appendices S8–S12. There were no differences in the model performance of regularization multiplier values for 2 m (*p* = 0.51) nonbrooding bobwhite first‐order models, and thus, a regularization multiplier value of 1 was used. Differences in regularization multiplier values existed for the 30 m bobwhite (*p* = 0.04), 2 m scaled quail (*p* = 0.02), and the 30 m scaled quail (*p* = 0.01) first‐order nonbrooding models, and regularization multiplier values of 4, 6, and 1.5 were used for these models, respectively. Based on average test omission errors, first‐order models for nonbrooding adults performed well for both bobwhite (2 m = 0.09, 30 m = 0.10) and scaled quail (2 m = 0.08, 30 m = 0.12).

**Figure 2:**
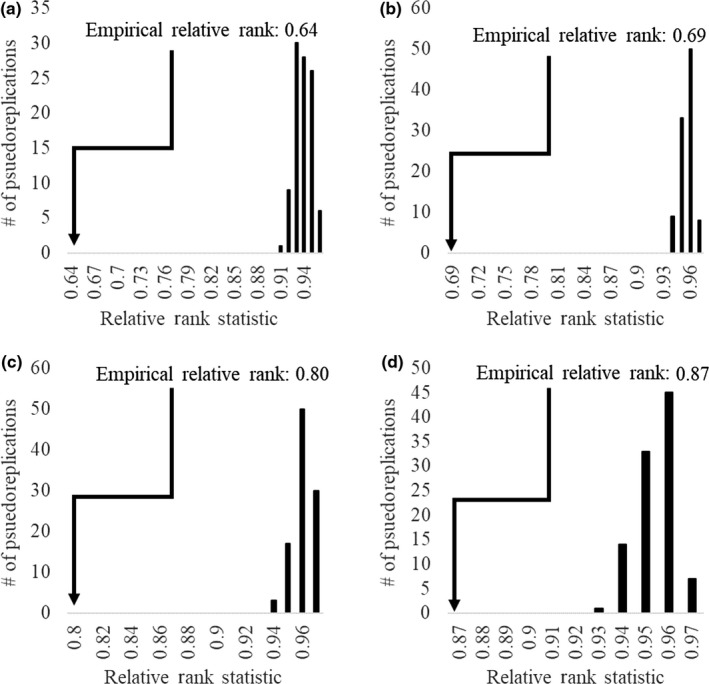
Null distributions of the relative rank metrics for northern bobwhite (*Colinus virginianus*; 2 m grain [A] and 30 m grain [B]) and scaled quail (*Callipepla squamata*; 2 m grain [C] and 30 m grain [D]) created from 100 pseudoreplications of Maxent models that compared randomly selected locations from brooding and nonbrooding adult locations collected during the breeding seasons from 2013 to 2014 at Beaver River WMA, Oklahoma, USA. Empirical relative ranks estimated from habitat suitability indices used in our analyses are indicated with a black arrow

A comparison of first‐order models for brooding versus nonbrooding bobwhite adults indicated relative rank values of 0.64 and 0.69 for the 2 m and 30 m models, respectively. For scaled quail, relative rank values of 0.80 and 0.87 were estimated for 2 m and 30 m models, respectively. Based on null distributions of relative rank values, all four empirical relative ranks comparing the similarity between brooding and nonbrooding habitat suitability models were statistically significant (Figure 2), indicating a divergence in space use by brooding adults compared to nonbrooding adults. However, bobwhite models indicated a much higher pattern of habitat use divergence by brooding adults, in which the empirical relative rank value was 0.27 units away from a null distribution for the 2 m models and 0.24 units away from the null distribution for the 30 m models (Figure 2). Conversely, empirical relative rank values for scaled quail models were 0.14 units away from the null distribution for 2 m models and 0.06 units away for the 30 m models (Figure 2). This suggests that there was a greater amount of similarity in space use between brooding and nonbrooding adults for scaled quail when compared to bobwhite.

### 3.3 Chick survival

Our baseline temporal survival models suggested no support for temporal survival trends for bobwhite chicks (null model AIC_c_ = 161.21, *w* = 0.28) and no support for temporal survival trends for scaled quail chicks (null model AIC_c_ = 171.59, *w* = 0.39). Overdispersion factors calculated from these baseline temporal models suggested that there was variance inflation due to a lack of independence between individuals for scaled quail chicks (*ĉ* = 1.54), whereas underdispersion and independence were evident for bobwhite chicks (*ĉ* = 0.52).

**Figure 3:**
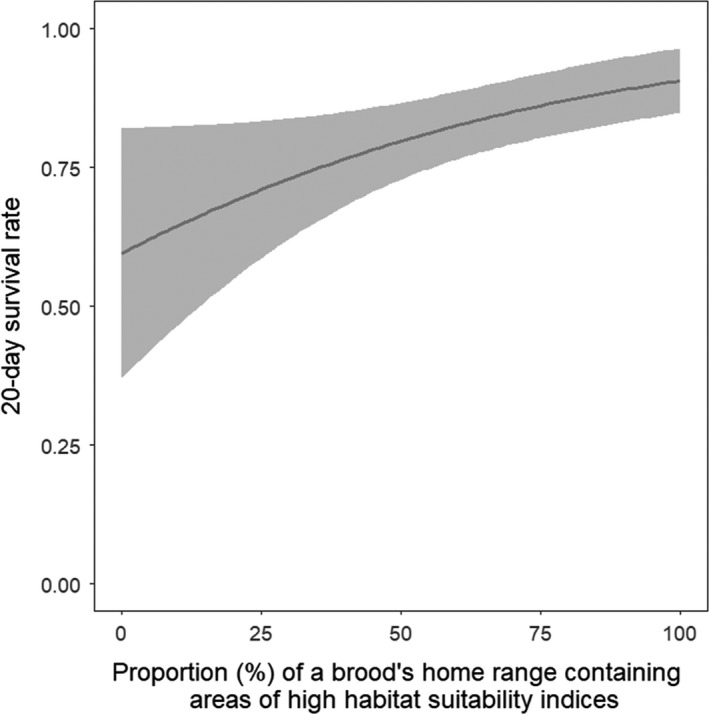
The 20‐day survival probability of northern bobwhite (*Colinus virginianus*) chicks as a function of the proportion of a brood's home range containing first‐order derived areas of high habitat suitability^a^ at the 30 m grain during the breeding seasons from 2013 to 2014 at Beaver River WMA, Oklahoma, USA. The solid line indicates survival probabilities, while the gray area indicates 95% confidence intervals^b^. ^a^Habitat suitability was estimated through the Maxent algorithm. ^b^The delta method was used to calculate 20‐day survival probabilities and confidence intervals from daily survival rates estimated from Program MARK

The most parsimonious survival model for bobwhite chicks was the univariate model with the variable representing the percentage of high habitat suitability values included within a brood's home range at the 30 m grain (*β* = 0.02, *SE* = 0.006, *w* = 0.41; Table 3). Though three other models had a ΔQAIC_c_ < 2, only the 30 m high suitability variable was considered significant based on confidence intervals not overlapping 0. Based on this model, daily survival rate (DSR) for bobwhite chicks was 0.9917 (*SE* = 0.0017), which extrapolated to an overall 20‐day survival rate of 0.8474 (*SE* = 0.0298). The beta estimate for 30 m high suitability values in this model indicated an increase in 20‐day survival rates for bobwhite chicks when a brood's home range had greater amounts of high HSI (Figure 3). The 20‐day survival rate for bobwhite chicks was estimated as 0.91 (*SE* = 0.03) when a brood's home range was composed of 100% of high habitat suitability areas and decreased to 0.60 (*SE* = 0.11) when it was comprised of 0%.

**Table 3:** Best performing models^a^ from the nest survival model in Program MARK estimating northern bobwhite (*Colinus virginianus*) chick survival from 2013 to 2014 at Beaver River WMA, Oklahoma, USA

Model	QAIC_c_	ΔQAIC_c_	*w* b	Model likelihood	*K* c	Qdeviance
30 m high probability^d^	300.09	0.00	0.36	1.00	2	296.09
30 m high probability + 2 m low probability	301.56	1.46	0.17	0.48	3	295.54
30 m high probability + 30 m low probability	301.75	1.66	0.16	0.44	3	295.73
PC2^e^ + 30 m high probability	301.86	1.77	0.15	0.41	3	295.84
Linear time	305.46	5.36	0.03	0.07	2	301.45
Null	305.50	5.41	0.02	0.07	1	303.50
2 m high probability	305.72	5.62	0.02	0.06	2	301.71
2 m low probability	306.24	6.15	0.02	0.05	2	302.23
Quadratic time	306.32	6.23	0.02	0.04	2	302.31
PC1^e^	306.79	6.69	0.01	0.04	2	302.78
Home range size (ha)	307.16	7.07	0.01	0.03	2	303.15
PC2	307.31	7.21	0.01	0.03	2	303.30
Ordinal date of hatch	307.40	7.31	0.01	0.03	2	303.39
30 m low probability	307.47	7.38	0.01	0.03	2	303.46

a Model performance was determined based on the lowest quasi‐Akaike's information criterion value corrected for small sample sizes (QAIC_c_). The model‐building strategy was based on guidelines created by Hosmer et al. (2013).

b Model weight.

c Number of parameters.

d High and low probability refer to metrics representing the percent of a brood's home range that is made up of high and low probability of habitat suitability values as described in Table 1.

e PC1 represented hot dry days, while PC2 represented cooler and more humid days with precipitation events (Appendix S2).

For scaled quail chick survival, we did not detect any direct impacts of space use on chick survival as the null model was the best supported model (*w* = 0.20; Table 4). Though eight univariate models had ΔQAIC_c_ < 2, all *β* coefficients for these parameters had confidence intervals overlapping 0. The overall DSR for scaled quail chicks was 0.9747 (*SE* = 0.0059), and the overall 20‐day survival rate (0.5993, *SE* = 0.0720) for scaled quail chicks was statistically lower than that of bobwhite chicks.

**Table 4:** Best performing models^a^ from the nest survival model in Program MARK estimating scaled quail (*Callipepla squamata*) chick survival from 2013 to 2014 at Beaver River WMA, Oklahoma, USA

Model	QAIC_c_	ΔQAIC_c_	*w* b	Model likelihood	*K* c	Qdeviance
Null	171.59	0.00	0.20	1.00	1	169.58
30 m low probability	172.62	1.03	0.12	0.60	2	168.61
Ordinal date of hatch	173.27	1.70	0.08	0.43	2	169.26
Home range size (ha)	173.36	1.77	0.08	0.41	2	169.35
2 m low probability	173.36	1.77	0.08	0.41	2	169.35
2 m high probability	173.46	1.90	0.08	0.39	2	169.47
Quadratic time	173.50	1.91	0.08	0.38	2	169.49
PC2^d^	173.56	1.97	0.07	0.37	2	169.55
Linear time	173.58	1.99	0.07	0.37	2	169.57
PC1^d^	173.59	2.00	0.07	0.37	2	169.58
30 m high probability	173.59	2.01	0.07	0.37	2	169.58

a Model performance was determined based on the lowest quasi‐Akaike's information criterion value corrected for small sample sizes (QAIC_c_). The model‐building strategy was based on guidelines created by Hosmer et al. (2013).

b Model weight.

c Number of parameters.

High and low probability refer to metrics representing the percent of a brood's home range that is made up of high and low probability of habitat suitability values as described in Table 1.

d PC1 represented hot dry days, while PC2 represented cooler and more humid days with precipitation events (Appendix S2).

### 3.4 Adult survival

**Figure 4:**
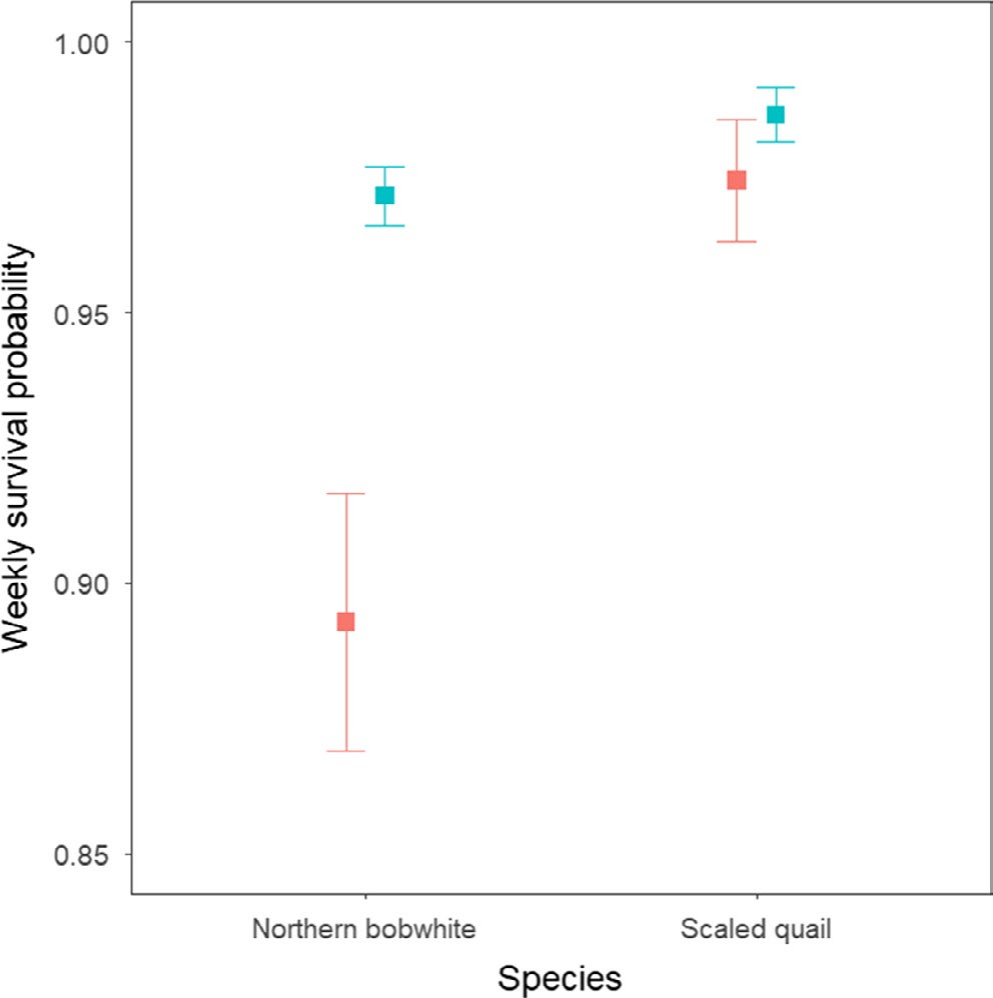
Estimated weekly survival probabilities for northern bobwhite (*Colinus virginianus*) and scaled quail (*Callipepla squamata*) adults based on their reproductive status (red = brooding, cyan = nonbrooding). Weekly survival probabilities were estimated during June 23–October 20 and June 9–October 12 for northern bobwhite and scaled quail, respectively, during the 2013–2014 breeding seasons at Beaver River WMA, Oklahoma, USA

A total of 187 bobwhite (39 brooding adults) and 114 scaled quail (31 brooding adults) were included in our adult survival analyses. The most parsimonious model for the northern bobwhite adult survival analysis indicated the reproductive status (i.e., either brooding or nonbrooding) of adults influenced survival probabilities (*β* = −1.38, *SE* = 0.31; Table 5). The estimated DSR was 0.9839 (*SE* = 0.0038) for brooding adults and was 0.9959 (*SE* = 0.0008) for nonbrooding adults. This resulted in weekly survival probabilities of 0.9716 (*SE* = 0.0054) and 0.8928 (*SE* = 0.0006) for nonbrooding and brooding adults, respectively (Figure 4). Conversely, there was no support for the reproductive status of an adult influencing adult survival for scaled quail as the null model was our best supported model (Table 5). The univariate model for reproductive status was included within a ΔAIC_c_ < 2. However, the *β* coefficient had confidence intervals that overlapped 0 (*β* = −0.65, *SE* = 0.59), and thus, this effect was not considered statistically significant (Figure 4). Based on the null model, the estimated DSR was 0.9976 (*SE* = 0.0007) for scaled quail which resulted in a weekly survival probability of 0.9832 (*SE* = 0.0048).

**Table 5:** Best performing models^a^ from nest survival models in Program MARK estimating survival of brooding and nonbrooding northern bobwhite (*Colinus virginianus*) and scaled quail adults from June 23–October 20 and June 9–October 12, respectively, during the 2013–2014 breeding seasons at Beaver River WMA, Oklahoma, USA

Model	AIC_c_	ΔAIC_c_	*w* b	Model likelihood	*K* c	Deviance
Northern bobwhite (*Colinus virginianus*)						
Brooding status^d^	454.44	0.00	0.99	1.00	2	450.44
Null	469.86	15.42	<0.01	<0.01	1	467.86
Time	470.58	16.14	<0.01	<0.01	2	466.58
Time^2^	470.94	16.50	0.00	0.00	2	466.94
Scaled quail (*Callipepla squamata*)						
Null	160.45	0.00	0.37	1.00	1	158.45
Brooding status^d^	161.28	0.83	0.25	0.66	2	157.28
Time^2^	161.79	1.34	0.19	0.51	2	157.79
Time	161.79	1.35	0.19	0.51	2	157.79

a Model performance was determined based on the lowest Akaike's information criterion value corrected for small sample sizes (AIC_c_).

b Model weight.

c Number of parameters.

d Brooding status indicates whether an adult individual was actively brooding during this period.

## 4 DISCUSSION

It is evident based on our results that adaptive decisions made by brooding adults can directly influence the survival of individual chicks, yet may come at a cost of increased parental risk for some species. Specifically, bobwhite chick survival was directly influenced by parental behavioral modifications that increased the amount of high HSIs within their home range, yet parental risk was greater for adults actively brooding. This likely represents a trade‐off between parental risk and offspring survival, in which bobwhite adults are selecting for areas that may directly influence chick survival at the risk to their own survival. Our results indicate that both bobwhite and scaled quail were behaviorally modifying their space use during the brood‐rearing stage when compared to nonbrooding adult space use. Such behavioral adaptations during the brooding period are common in other Galliformes (Dinkins, Conover, Kirol, & Beck, 2012; Gibson et al., 2017; Mangelinckx et al., 2018; Zhao et al., 2018), and previous research has indicated an evident pattern of altered space use for brooding bobwhite (Carroll et al., 2015). However, the degree to which individuals modify their space use during this period can vary across space, time, and species. For instance, we found a greater degree of altered space use for brooding bobwhite when compared to brooding scaled quail (Figure 2). Though life‐history theory would suggest that both species should be expected to incur a cost due to these behavioral changes, we observed clear divergent rates of alternative reproductive strategies between species during the same time period (Davis et al., 2017), suggesting that interspecific differences in brooding strategies may be expected in sympatric species. Such relationships offer important insight into the conservation of these species during a vulnerable and important life‐history stage.

Life‐history theory suggests that there should be a trade‐off between parental risk and offspring success for short‐lived species (Ghalambor & Martin, 2001; Stearns, 1976). However, direct offspring success to such behavioral modifications from attending parents may be hard to quantify due to logistic constraints associated with marking individuals at such a vulnerable period, particularly for precocial species. By integrating both chick survival and adult survival simultaneously into our analyses, we attempted to eliminate any unobserved components of such a risk‐to‐reproduction trade‐off associated with these species. Such unobserved components have been posited as a potential source of ambiguity associated with studies assessing how adaptive habitat selection influences reproductive success and overall fitness (Bloom et al., 2013; Uboni et al., 2017). Though we attempted to eliminate these unobserved components (by assessing chick and adult survival simultaneously), our study suggests the potential for continued ambiguity in this relationship across sympatric species. This is because we observed a direct influence of parental habitat selection on bobwhite chick survival at the cost of decreased survival for the attendant parents, whereas no relationships were detected for scaled quail. Though a smaller sample size associated with scaled quail chicks and adults may have precluded the detection of such a relationship, other possibilities exist that could explain this null relationship. A lack of significant effects of brooding on parental fitness may occur if higher‐quality individuals (i.e., those with more experience or genetic benefits) are inherently more likely to engage in reproductive activities (Arnold, Roche, Devries, & Howerter, 2012; Cam, Link, Cooch, Monnat, & Danchin, 2002) or if a species is more likely to allocate resources for self‐maintenance. For instance, it has been suggested that scaled quail have adapted to arid and semi‐arid regions (such as our study area) by allocating more resources toward self‐maintenance rather than toward reproductive output whereas the opposite is true for bobwhite because they evolved under more mesic conditions (Giuliano, Patiño, & Lutz, 1998). Likewise, the null relationship between HSI and scaled quail chick survival could be driven by relationships in which environmental conditions influencing both species' occurrence and species' survival are disparate from the conditions only influencing a species' occurrence (Bacon et al., 2016; Ficetola, Miaud, Pompanon, & Taberlet, 2008). Furthermore, this null relationship could be related to the spatial scales that were used in our analyses (Bloom et al., 2013), though we attempted to account for this by using two grain sizes representing fine (2 m) and coarse (30 m) habitat configurations.

The scale at which organisms respond to environmental conditions and/or resources should vary across space and time if habitat selection is an adaptive process (Bloom et al., 2013; Wiens, 1989). Furthermore, variation in the scale at which organisms select habitat will also change during different life‐history stages (Addicott et al., 1987; Chalfoun & Martin, 2007; McGarigal et al., 2016) which could potentially decouple the link between space use and demographic parameters (Chalfoun & Martin, 2007; Robertson & Hutto, 2006). For instance, if the second‐order selection (i.e., selection of home ranges [Johnson, 1980]) for an individual is constrained during a specific life‐history stage such as brood rearing (Carroll et al., 2015) due to decreased movement abilities or physiological constraints, a brood‐rearing adult may not be able to select certain areas on a landscape that increases brood survival. We found that the brooding habitat selection–fitness relationship is dynamic across spatial grains, as the relationship between bobwhite chick survival and HSI was only significant at the 30 m grain. In this study, we used an index of habitat suitability derived from occurrence locations (through radiotelemetry) and a Maxent algorithm to determine how intraspecific space‐use decisions of brooding adults influenced chick survival relative to what was available at the landscape level. Though this may not be a direct measure of habitat quality per se (such as a quantified measure of food resources), this metric does predict the probability of a target species occurring in an area based on the environmental conditions considered in a model and is commonly used as a tool for conservation purposes (Guisan & Thuiller, 2005). However, the relationship between habitat suitability and demographic parameters related to population persistence is often complicated and disparate across species, space, and time (Bacon et al., 2016). Specifically, our results suggest that the relationship between habitat suitability and chick survival can vary across species and spatial scale. Therefore, if such indices are to be used as a tool for conservation efforts, single‐scale suitability indices may be too simple and miss such complex relationships (Bacon et al., 2016).

Our research illustrates the importance in understanding the complex relationships between parental space use, habitat suitability, and how these interact to influence chick and attending parental survival. It is evident that these relationships exist in a complex state that can be species‐specific, spatially variable, and potentially influenced by life‐history strategies. Indeed, for bobwhite in our study, there was a clear cost of parental attendance in exchange for offspring survival, which can help explain other aspects of their life‐history strategies. For example, brood amalgamation on our study site was more common in bobwhite broods than scaled quail broods at the chick life stage (Orange, 2015). Such breeding behavioral strategies have been postulated to offer survival benefits for the offspring (Dahlgren, Messmer, & Koons, 2010; Eadie, Kehoe, & Nudds, 1988; Lott & Mastrup, 1999), yet could have implications into understanding a link between demographics and differential habitat selection. Furthermore, when assessing demographic parameters such as precocial chick survival, these relationships are hierarchical in that it is ultimately tied to the brooding adult's behavioral adaptations. Evidence emphasizes the importance of chick survival for population persistence of *r*‐selected species (Burger, Dailey, Kurzejeski, & Ryan, 1995; Colwell et al., 2007; Ludwig, Aebischer, Bubb, Roos, & Baines, 2018; Sandercock et al., 2008; Terhune, Sission, Grand, & Stribling, 2007), and if concepts such as habitat selection and habitat suitability are to be used for conservation purposes (Morrison et al., 2012), efforts to understand simultaneous trade‐offs between offspring survival, adaptive habitat selection, and parental risk must be considered.

## CONFLICT OF INTEREST

The authors declare no conflict of interest.

## AUTHOR CONTRIBUTIONS

All authors contributed equally to this manuscript.

## DATA AVAILABILITY STATEMENT

Data available from the Dryad Digital Repository: https://doi.org/10.5061/dryad.2ct6558.

## References

[ece35472-bib-0001] Ackerman, J. T. , Herzog, M. P. , Takekawa, J. Y. , & Hartman, C. A. (2014). Comparative reproductive biology of sympatric species: Nest and chick survival of American avocets and black‐necked stilts. Journal of Avian Biology, 45, 609–623. 10.1111/jav.00385

[ece35472-bib-0002] Addicott, J. F. , Aho, J. M. , Antolin, M. F. , Padilla, D. K. , Richardson, J. S. , & Soluk, D. A. (1987). Ecological neighborhoods: Scaling environmental patterns. Oikos, 49, 340–346. 10.2307/3565770

[ece35472-bib-0003] Aldridge, C. L. , & Boyce, M. S. (2007). Linking occurrence and fitness to persistence: Habitat‐based approach for endangered greater sage‐grouse. Ecological Applications, 17, 508–526. 10.1890/05-1871 17489256

[ece35472-bib-0004] Andes, A. K. , Buckley, B. R. , Warren, T. L. , Woods, P. C. , Yancey, S. R. , & Dabbert, C. B. (2012). Use of a thermal camera to aid in capturing northern bobwhite quail chicks. Wildlife Society Bulletin, 36, 371–375. 10.1002/wsb.141

[ece35472-bib-0005] Araújo, C. B. , Marcondes‐Machado, L. O. , & Costa, G. C. (2014). The importance of biotic interactions in species distribution models: A test of the Eltonian noise hypothesis using parrots. Journal of Biogeography, 41, 513–523. 10.1111/jbi.12234

[ece35472-bib-0006] Arnold, T. W. , Roche, E. A. , Devries, J. H. , & Howerter, D. W. (2012). Costs of reproduction in breeding female mallards: Predation risk during incubation drives annual mortality. Avian Conservation and Ecology, 7, 1 10.5751/ACE-00504-070101

[ece35472-bib-0007] Baasch, D. M. , Tyre, A. J. , Millspaugh, J. J. , Hygnstrom, S. E. , & Vercauteren, K. C. (2010). An evaluation of three statistical methods used to model resource selection. Ecological Modelling, 221, 565–574. 10.1016/j.ecolmodel.2009.10.033

[ece35472-bib-0008] Bacon, L. , Hingrat, Y. , Jiguet, F. , Monnet, A.‐C. , Sarrazin, F. , & Robert, A. (2016). Habitat suitability and demography, a time‐dependent relationship. Ecology and Evolution, 7, 2214–2222. 10.1002/ece3.2821 PMC5383465 28405285

[ece35472-bib-0009] Baldwin, R. A. (2009). Use of maximum entropy modeling in wildlife research. Entropy, 11, 854–866. 10.3390/e11040854

[ece35472-bib-0010] Beerens, J. M. , Frederick, P. C. , Noonburg, E. G. , & Gawlik, D. E. (2015). Determining habitat quality for species that demonstrate dynamic habitat selection. Ecology and Evolution, 5, 5685–5697. 10.1002/ece3.1813 27069617 PMC4813099

[ece35472-bib-0011] Bellrose, F. C. , & Holm, D. J. (1994). Ecology and management of the wood duck. Mechanicsburg, PA: Stackpole Books.

[ece35472-bib-0012] Beyer, H. L. (2012). Geospatial modelling environment (Version 0.7.2.1). Retrieved from http://www.spatialecology.com/gme

[ece35472-bib-0013] Bishop, C. J. , White, G. C. , & Lukacs, P. M. (2008). Evaluating dependence among mule deer siblings in fetal and neonatal survival analyses. Journal of Wildlife Management, 72, 1085–1093. 10.2193/2007-423

[ece35472-bib-0014] Blomberg, E. J. , Sedinger, J. S. , Nonne, D. V. , & Atamian, M. T. (2013). Seasonal reproductive costs contribute to reduced survival of female greater sage‐grouse. Journal of Avian Biology, 44, 149–158. 10.1111/j.1600-048X.2012.00013.x

[ece35472-bib-0015] Bloom, P. M. , Clark, R. G. , Howerter, D. W. , & Armstrong, L. M. (2013). Multi‐scale habitat selection affects offspring survival in a precocial species. Oecologia, 173, 1249–1259. 10.1007/s00442-013-2698-4 23843036

[ece35472-bib-0016] Bock, C. E. , & Jones, Z. F. (2004). Avian habitat evaluation: Should counting birds count? Frontiers in Ecology and the Environment, 2, 403–410. 10.1890/1540-9295(2004)002[0403:AHESCB]2.0.CO;2

[ece35472-bib-0017] Brock, F. V. , Crawford, K. C. , Elliot, R. L. , Cuperus, G. W. , Stadler, S. J. , Johnson, H. L. , & Eilts, M. D. (1995). The Oklahoma Mesonet, a technical overview. Journal of Atmospheric and Oceanic Technology, 12, 5–19.

[ece35472-bib-0018] Brøseth, H. , & Pedersen, H. C. (2010). Disturbance effects of hunting activity in a willow ptarmigan *Lagopus lagopus* population. Wildlife Biology, 16, 241–248. 10.2981/09-096

[ece35472-bib-0019] Burger, L. W. , Dailey, T. V. , Kurzejeski, E. , & Ryan, M. R. (1995). Seasonal and annual survival and cause specific mortality of northern bobwhite in Missouri. Journal of Wildlife Management, 59, 401–410. 10.2307/3808954

[ece35472-bib-0020] Burkepile, N. A. , Conelly, J. W. , Stanley, D. W. , & Reese, K. P. (2002). Attachment of radiotransmitters to one‐day‐old sage grouse chicks. Wildlife Society Bulletin, 30, 93–96.

[ece35472-bib-0021] Burnham, K. P. , & Anderson, D. R. (2002). Model selection and multimodel inference: A practical information theoretic approach. New York, NY: Springer‐Verlag.

[ece35472-bib-0022] Cam, E. , Link, W. A. , Cooch, E. G. , Monnat, J. Y. , & Danchin, E. (2002). Individual covariation in life‐history traits: Seeing the trees despite the forest. American Naturalist, 159, 96 10.2307/3079316 18707403

[ece35472-bib-0023] Carroll, J. M. , Davis, C. A. , Elmore, R. D. , Fuhlendorf, S. D. , & Thacker, E. T. (2015). Thermal patterns constrain diurnal behavior of a ground‐dwelling bird. Ecosphere, 6, 1–15. 10.1890/ES15-00163.1

[ece35472-bib-0024] Caudill, D. , Guttery, M. R. , Bibles, B. , Messmer, T. A. , Caudill, G. , Leone, E. , … Chi, R. (2014). Effects of climatic variation and reproductive trade‐offs vary by measure of reproductive effort in greater sage‐grouse. Ecosphere, 5, 154 10.1890/ES14-00124.1

[ece35472-bib-0025] Chalfoun, A. D. , & Martin, T. E. (2007). Assessments of habitat preferences and quality depend on spatial scale and metrics of fitness. Journal of Applied Ecology, 44, 983–992. 10.1111/j.1365-2664.2007.01352.x

[ece35472-bib-0026] Chernick, M. R. (1999). Bootstrap methods: A practitioner's guide. New York, NY: Wiley Series in Probability Statistics.

[ece35472-bib-0027] Colwell, M. A. , Hurley, S. J. , Hall, J. N. , & Dinsmore, S. J. (2007). Age‐related survival and behavior of snowy plover chicks. Condor, 109, 638–647. 10.1650/8236.1

[ece35472-bib-0028] Cooch, E. G. , & White, G. C. (2017). Program MARK: A gentle introduction (17th ed.). Retrieved from http://www.phidot.org/software/mark/docs/book/

[ece35472-bib-0029] Dahlgren, D. K. , Messmer, T. A. , & Koons, D. N. (2010). Achieving better estimates of greater sage‐grouse chick survival in Utah. Journal of Wildlife Management, 74, 1286–1294. 10.2193/2009-093

[ece35472-bib-0030] Davis, C. A. , Orange, J. P. , Van Den Bussche, R. A. , Elmore, R. D. , Fuhlendorf, S. D. , Carroll, J. M. , … Leslie, D. M. Jr (2017). Extrapair paternity and nest parasitism in two sympatric quail. The Auk, 134, 811–820. 10.1642/AUK-16-162.1

[ece35472-bib-0031] Dawson, A. , Hinsley, S. , Ferns, P. , Bonser, R. C. , & Eccleston, L. (2000). Rate of moult affects feather quality: a mechanism linking current reproductive effort to future survival. Proceedings of the Royal Society B: Biological Sciences, 267(1457), 2093–2098. 10.1098/rspb.2000.1254 PMC1690790 11416914

[ece35472-bib-0032] Dinkins, J. B. , Conover, M. R. , Kirol, C. P. , & Beck, J. L. (2012). Greater sage‐grouse (*Centrocercus urophasianus*) select nest sites and brood sites away from avian predators. The Auk, 129, 600–610.

[ece35472-bib-0033] Dinsmore, S. J. , White, G. C. , & Knopf, F. L. (2002). Advanced techniques for modeling avian nest survival. Ecology, 83, 3476–3488. 10.1890/0012-9658(2002)083[3476:ATFMAN]2.0.CO;2

[ece35472-bib-0034] Donovan, T. M. , & Thompson, F. R. (2001). Modeling the ecological trap hypothesis: A habitat and demographic analysis for migrant songbirds. Ecological Applications, 11, 871–882. 10.1890/1051-0761(2001)011[0871:MTETHA]2.0.CO;2

[ece35472-bib-0035] Dormann, C. F. , Elith, J. , Bacher, S. , Buchmann, C. , Carl, G. , Carŕe, G. , … Lautenbach, S. (2013). Collinearity: A review of methods to deal with it and a simulation study evaluating their performance. Ecography, 36, 27–46. 10.1111/j.1600-0587.2012.07348.x

[ece35472-bib-0036] Dreitz, V. J. (2009). Parental behavior of a precocial species: Implications for juvenile survival. Journal of Applied Ecology, 46, 870–878. 19946600 10.1111/j.1365-2664.2009.01658.x PMC2779466

[ece35472-bib-0037] Dreitz, V. J. , Baeten, L. A. , Davis, T. , & Riordan, M. M. (2011). Testing radiotransmitter attachment techniques on northern bobwhite and chukar chicks. Wildlife Society Bulletin, 35, 475–480. 10.1002/wsb.73

[ece35472-bib-0038] Eadie, J. , Kehoe, F. P. , & Nudds, T. D. (1988). Pre‐hatch and post‐hatch brood amalgamation in North American anatidae: A review of hypotheses. Canadian Journal of Zoology, 66, 1709–1721. 10.1139/z88-247

[ece35472-bib-0039] Elith, J. , Phillips, S. J. , Hastie, T. , Dudik, M. , Chee, Y. E. , & Yates, C. J. (2011). A statistical explanation of MaxEnt for ecologists. Diversity and Distributions, 17, 43–57. 10.1111/j.1472-4642.2010.00725.x

[ece35472-bib-0040] ESRI (2011). ArcGIS Desktop: Release 10. Redlands, CA: Environmental Systems Research Institute.

[ece35472-bib-0041] Ficetola, G. F. , Miaud, C. , Pompanon, F. , & Taberlet, P. (2008). Species detection using environmental DNA from water samples. Biology Letters, 4, 423–425. 10.1098/rsbl.2008.0118 18400683 PMC2610135

[ece35472-bib-0042] Foley, D. H. , Rueda, L. M. , Peterson, A. T. , & Wilkerson, R. C. (2008). Potential distribution of two species in the medically important *Anopheles minimus* complex (Diptera: Culicidae). Journal of Medical Entomology, 45, 852–860. 10.1093/jmedent/45.5.852 18826026

[ece35472-bib-0043] Folmer, E. O. , & Piersma, T. (2012). The contribution of resource availability and social forces to foraging distributions: A spatial lag modelling approach. Animal Behavior, 84, 1371–1380.

[ece35472-bib-0044] Garrick, E. J. , Amundson, C. L. , & Seddon, P. J. (2017). Duckling survival of mallards in Southland, New Zealand. Journal of Wildlife Management, 81, 858–867.

[ece35472-bib-0045] Gates, J. E. , & Gysel, L. W. (1978). Avian nest dispersion and fledging success in field‐forest ecotones. Ecology, 59, 871–883. 10.2307/1938540

[ece35472-bib-0046] Ghalambor, C. K. , & Martin, T. E. (2001). Fecundity‐survival trade‐offs and parental risk‐taking in birds. Science, 292, 494–497. 10.1126/science.1059379 11313493

[ece35472-bib-0047] Gherghel, I. , Brischoux, F. , & Papeş, M. (2018). Using biotic interactions in broad‐scale estimates of species' distributions. Journal of Biogeography, 45, 2216–2225. 10.1111/jbi.13361

[ece35472-bib-0048] Gibson, D. , Blomberg, E. J. , Atamian, M. T. , & Sedinger, J. S. (2017). Weather, habitat composition, and female behavior interact to modify offspring survival in Greater Sage‐Grouse. Ecological Applications, 27, 168–181. 28052504 10.1002/eap.1427

[ece35472-bib-0049] Giuliano, W. M. , Patiño, R. , & Lutz, R. S. (1998). Comparative reproductive and physiological responses of northern bobwhite and scaled quail to water deprivation. Comparative Biochemistry and Physiology Part A: Molecular & Integrative Physiology, 119, 781–786. 10.1016/S1095-6433(98)01015-0

[ece35472-bib-0050] Gregg, M. A. , & Crawford, J. A. (2009). Survival of greater sage‐grouse chicks and broods in the northern Great Basin. Journal of Wildlife Management, 73, 904–913. 10.2193/2007-410

[ece35472-bib-0051] Guisan, A. , & Thuiller, W. (2005). Predicting species distribution: Offering more than simple habitat models. Ecology Letter, 8, 993–1009. 10.1111/j.1461-0248.2005.00792.x 34517687

[ece35472-bib-0052] Hagen, C. A. , Pitman, J. C. , Sandercock, B. K. , Robel, R. J. , & Applegate, R. D. (2011). Age‐specific survival and probable causes of mortality in lesser prairie‐chickens. Journal of Wildlife Management, 71, 518–525.

[ece35472-bib-0053] Hastie, T. , Tibshirani, R. , & Friedman, J. (2001). The elements of statistical learning. New York, NY: Springer, New York Inc.

[ece35472-bib-0054] Hosmer, J. , David, W. , Lemeshow, S. , & Sturdivant, R. X. (2013). Model‐building strategies and methods for logistic regression In Applied logistic regression (pp. 89–151). Hoboken, NJ: John Wiley and Sons Inc.

[ece35472-bib-0055] Jackson, D. A. (1993). Stopping rules in principal components analysis: A comparison of heuristical and statistical approaches. Ecology, 74, 2204–2214. 10.2307/1939574

[ece35472-bib-0056] Jenks, G. F. (1967). The data model concept in statistical mapping. International Yearbook of Cartography, 7, 186–190.

[ece35472-bib-0057] Johnson, D. (1980). The comparison of usage and availability measurements for evaluating resource preference. Ecology, 61, 65–71. 10.2307/1937156

[ece35472-bib-0058] Kolbe, J. J. , & Janzen, F. J. (2002). Impact of nest‐site selection on nest success and nest temperature in natural and disturbed habitats. Ecology, 83, 269–281. 10.1890/0012-9658(2002)083[0269:IONSSO]2.0.CO;2

[ece35472-bib-0059] Koons, D. , & Rotella, J. (2003). Comparative nesting success of sympatric lesser scaup and ring‐necked ducks. Journal of Field Ornithology, 74, 222–229. 10.1648/0273-8570-74.3.222

[ece35472-bib-0060] Kristan, W. B. (2003). The role of habitat selection behavior in population dynamics: Source‐sink systems and ecological traps. Oikos, 103, 457–468. 10.1034/j.1600-0706.2003.12192.x

[ece35472-bib-0061] Lack, D. (1933). Habitat selection in birds. With special reference to the effects of afforestation on the Breckland avifauna. Journal of Animal Ecology, 2, 239–262. 10.2307/961

[ece35472-bib-0062] Larson, M. A. , Clark, M. E. , & Winterstein, S. R. (2001). Survival of ruffed grouse chicks in northern Michigan. Journal of Wildlife Management, 65, 880–886. 10.2307/3803037

[ece35472-bib-0063] Lengyel, S. (2006). Spatial differences in breeding success in the pied avocet *Recurvirostra avosetta*: Effects of habitat on hatching success and chick survival. Journal of Avian Biology, 37, 381–395. 10.1111/j.0908-8857.2006.03501.x

[ece35472-bib-0064] Liu, C. , White, M. , & Newell, G. (2013). Selecting thresholds for the prediction of species occurrence with presence‐only data. Journal of Biogeography, 40, 778–789. 10.1111/jbi.12058

[ece35472-bib-0065] Lohr, M. , Collins, B. M. , Williams, C. K. , & Castelli, P. M. (2011). Life on the edge: Northern bobwhite ecology at the northern periphery of their range. Journal of Wildlife Management, 75, 52–60. 10.1002/jwmg.25

[ece35472-bib-0066] Lott, D. F. , & Mastrup, S. N. K. (1999). Facultative communal brood rearing in California quail. Condor, 101, 678–681. 10.2307/1370200

[ece35472-bib-0067] Ludwig, S. C. , Aebischer, N. J. , Bubb, D. , Roos, S. , & Baines, D. (2018). Survival of chicks and adults explains variation in population growth in a recovering red grouse Lagopus lagopus scotica population. Wildlife Biology, 2018: wlb.00430 10.2981/wlb.00430

[ece35472-bib-0068] Lusk, J. J. , Guthery, F. S. , Cox, S. A. , De Maso, S. J. , & Peoples, A. D. (2005). Survival and growth of northern bobwhite chicks in western Oklahoma. American Midland Naturalist, 153, 389–395. 10.1674/0003-0031(2005)153[0389:SAGONB]2.0.CO;2

[ece35472-bib-0069] Lustig, A. , Stouffer, D. B. , Roigé, M. , & Worner, S. P. (2015). Towards more predictable and consistent landscape metrics across spatial scales. Ecological Indicators, 57, 11–21. 10.1016/j.ecolind.2015.03.042

[ece35472-bib-0070] Mangelinckx, J. M. , Davis, S. R. B. , Allen, R. B. , Sullivan, K. , & Blomberg, R. J. (2018). Summertime resource selection and reproductive effects on survival of ruffed grouse. The Auk, 135, 933–948. 10.1642/AUK-17-212.1

[ece35472-bib-0071] Manly, B. F. J. , McDonald, L. L. , Thomas, D. L. , McDonald, T. L. , & Erickson, W. P. (2002). Resource selection by animals: Statistical design and analysis for field studies (2nd ed.). Dordrecht, The Netherlands: Kluwer Academic.

[ece35472-bib-0072] Mathews, T. W. , Tyre, A. J. , Taylor, J. S. , Lusk, J. J. , & Powell, L. A. (2011). Habitat selection and brood survival of greater prairie‐chickens In Sandercock B. K., Martin K., & Segelbacher G. (Eds.). Ecology, conservation, and management of grouse. Studies in avian Biology (no. 39) (pp. 179–191). Berkeley, CA: University of California Press.

[ece35472-bib-0073] McGarigal, K. , Cushman, S. A. , & Ene, E. (2012). FRAGSTATS v4: Spatial pattern analysis program for categorical and continuous maps. Computer software program produced by the authors at the University of Massachusetts, Amherst. Retrieved from http://www.umass.edu/landeco/research/fragstats/fragstats.hmtl

[ece35472-bib-0074] McGarigal, K. , Wan, H. Y. , Zeller, K. A. , Timm, B. C. , & Cushman, S. A. (2016). Multi‐scale habitat selection modeling: A review and outlook. Landscape Ecology, 31, 1161–1175. 10.1007/s10980-016-0374-x

[ece35472-bib-0075] McPherson, R. A. , Fiebrich, C. A. , Crawford, K. C. , Kilby, J. R. , Grimsley, D. L. , Martinez, J. E. , … Sutherland, A. J. (2007). Statewide monitoring of the mesoscale environment: A technical update on the Oklahoma Mesonet. Journal of Atmospheric and Oceanic Technology, 24, 301–321. 10.1175/JTECH1976.1

[ece35472-bib-0076] Morrison, M. L. , Marcot, B. , & Mannan, W. (2012). Wildlife‐habitat relationships: Concepts and applications. Washington, DC: Island Press.

[ece35472-bib-0077] O'Neill, R. V. , Krummel, J. R. , Gardner, R. H. , Sugihara, G. , Jackson, B. , De Angelis, D. L. , … Graham, R. L. (1988). Indices of landscape pattern. Landscape Ecology, 1, 153–162. 10.1007/BF00162741

[ece35472-bib-0078] Orange, J. P. (2015). Breeding behavior, brood habitat use, and chick survival of two sympatric quail species at the periphery of their distributions. Master's thesis, Oklahoma State University, Stillwater, OK.

[ece35472-bib-0079] Orange, J. P. , Davis, C. A. , Elmore, R. D. , Tanner, E. P. , Fuhlendorf, S. D. , & Thacker, E. T. (2016). Evaluating the efficacy of brood flush counts: A case study in two quail species. Western North American Naturalist, 76, 485–492. 10.3398/064.076.0409

[ece35472-bib-0080] Peters, D. C. , Brooke, J. M. , Tanner, E. P. , Unger, A. M. , Keyser, P. D. , Harper, C. A. , … Morgan, J. J. (2015). Impact of experimental habitat manipulation on northern bobwhite survival. Journal of Wildlife Management, 79, 605–617. 10.1002/jwmg.873

[ece35472-bib-0081] Phillips, S. J. , Anderson, R. P. , & Schapire, R. E. (2006). Maximum entropy modeling of species geographic distributions. Ecological Modelling, 190, 231–259. 10.1016/j.ecolmodel.2005.03.026

[ece35472-bib-0082] Phillips, S. J. , & Dudík, M. (2008). Modeling of species distributions with Maxent: New extensions and a comprehensive evaluation. Ecography, 31, 161–175. 10.1111/j.0906-7590.2008.5203.x

[ece35472-bib-0083] Powell, L. A. (2007). Approximating variance of demographic parameters using the delta method: A reference for avian biologists. Condor, 109, 949–954. 10.1650/0010-5422(2007)109[949:AVODPU]2.0.CO;2

[ece35472-bib-0084] Radosavljevic, A. , & Anderson, R. P. (2014). Making better Maxent models of species distributions: Complexity, overfitting and evaluation. Journal of Biogeography, 41, 629–643.

[ece35472-bib-0085] Reznick, D. (1985). Costs of reproduction: An evaluation of the empirical evidence. Oikos, 44, 257–267. 10.2307/3544698

[ece35472-bib-0086] Ritters, K. H. , O'Neill, R. V. , Hunsaker, C. T. , Wickham, J. D. , Yankee, D. H. , Timmins, S. P. , … Jackson, B. L. (1995). A factor analysis of landscape pattern and structure metrics. Landscape Ecology, 10(1), 23–39. 10.1007/BF00158551

[ece35472-bib-0087] Robertson, B. A. , & Hutto, R. L. (2006). A framework for understanding ecological traps and an evaluation of existing evidence. Ecology, 87, 1075–1085. 10.1890/0012-9658(2006)87[1075:AFFUET]2.0.CO;2 16761584

[ece35472-bib-0088] Rollins, D. (2000). Status, ecology and management of scaled quail in West Texas. Proceedings of the National Quail Symposium, 4, 165–172.

[ece35472-bib-0089] Sahlean, T. C. , Gherghel, I. , Papeş, M. , Strugariu, A. , & Zamfirescu, Ş. R. (2014). Refining climate change projections for organisms with low dispersal abilities: A case study of the Caspian whip snake. PLoS ONE, 9, e91994 10.1371/journal.pone.0091994 24670422 PMC3966777

[ece35472-bib-0090] Sandercock, B. K. , Jensen, W. E. , Williams, C. K. , & Applegate, R. D. (2008). Demographic sensitivity of population change in northern bobwhite. Journal of Wildlife Management, 72, 970–982. 10.2193/2007-124

[ece35472-bib-0091] Schmutz, J. A. , Ward, D. H. , Sedinger, J. S. , & Rexstad, E. A. (1995). Survival estimation and the effects of dependency among animals. Journal of Applied Statistics, 22, 673–681. 10.1080/02664769524531

[ece35472-bib-0092] Seaman, D. E. , & Powell, R. A. (1996). An evaluation of accuracy of kernel density estimators for home range analysis. Ecology, 77, 2075–2085.

[ece35472-bib-0093] Sieving, K. (1992). Nest predation and differential insular extinction among selected forest birds of central Panama. Ecology, 73, 2310–2328. 10.2307/1941477

[ece35472-bib-0094] Smith, M. D. , Hammond, A. D. , Burger, L. W. Jr , Palmer, W. E. , Carver, A. V. , & Wellendorf, S. D. (2003). A technique for capturing northern bobwhite chicks. Wildlife Society Bulletin, 31, 1054–1060.

[ece35472-bib-0095] Stearns, S. C. (1976). Life‐history tactics: A review of the ideas. The Quarterly Review of Biology, 51, 3–47. 10.1086/409052 778893

[ece35472-bib-0096] Storch, D. , & Frynta, D. (1999). Evolution of habitat selection: Stochastic acquisition of cognitive clues? Evolutionary Ecology, 13, 591–600. 10.1023/A:1006765422475

[ece35472-bib-0097] Tanner, E. P. , Elmore, R. D. , Davis, C. A. , Fuhlendorf, S. D. , Dahlgren, D. K. , Thacker, E. T. , & Orange, J. P. (2016). Does the presence of oil and gas infrastructure potentially increase risk of harvest in northern bobwhite? Wildlife Biology, 22, 294–304. 10.2981/wlb.00254

[ece35472-bib-0098] Tanner, E. P. , Elmore, R. D. , Fuhlendorf, S. D. , Davis, C. A. , Dahlgren, D. K. , & Orange, J. P. (2017). Extreme climatic events constrain space use and survival of a ground‐nesting bird. Global Change Biology, 23, 1832–1846. 10.1111/gcb.13505 27633847

[ece35472-bib-0099] Tanner, E. P. , Elmore, R. D. , Fuhlendorf, S. D. , Davis, C. A. , Thacker, E. T. , & Dahlgren, D. K. (2015). Behavioral responses at distribution extremes: How artificial surface water can affect quail movement patterns. Rangeland Ecology and Management, 68, 476–484. 10.1016/j.rama.2015.07.008

[ece35472-bib-0100] Tanner, E. P. , Papeş, M. , Elmore, R. D. , Fuhlendorf, S. D. , & Davis, C. A. (2017). Incorporating abundance information and guiding variable selection for climate‐based ensemble forecasting of species' distributional shifts. PLoS ONE, 12, e0184316 10.1371/journal.pone.0184316 28886075 PMC5590900

[ece35472-bib-0101] Taylor, J. S. , Church, K. E. , & Rusch, D. H. (1999). Microhabitat selection by nesting and brood‐rearing northern bobwhite in Kansas. Journal of Wildlife Management, 63, 686–694. 10.2307/3802658

[ece35472-bib-0102] Taylor, J. S. , & Guthery, F. S. (1994). Components of northern bobwhite brood habitat in southern Texas. Southwestern Naturalist, 39, 73–77. 10.2307/3672196

[ece35472-bib-0103] Terhune, T. M. , Palmer, W. E. , & Wellendorf, S. D. (2019). Northern bobwhite chick survival and effects of weather. The Journal of Wildlife Management, 83(4), 963–974. 10.1002/jwmg.21655

[ece35472-bib-0104] Terhune, T. M. , Sission, D. C. , Grand, J. B. , & Stribling, H. L. (2007). Factors influencing survival of radiotagged and banded northern bobwhites in Georgia. Journal of Wildlife Management, 71, 1288–1297. 10.2193/2005-640

[ece35472-bib-0105] Uboni, A. , Smith, D. W. , Stahler, D. R. , & Vucetich, J. A. (2017). Selecting habitat to what purpose? The advantage of exploring the habitat‐fitness relationship. Ecosphere, 8, e01705 10.1002/ecs2.1705

[ece35472-bib-0106] van Horne, B. (1983). Density as a misleading indicator of habitat quality. Journal of Wildlife Management, 47, 893–901. 10.2307/3808148

[ece35472-bib-0107] Varo, N. (2008). Breeding biology of two sympatric coots with contrasting conservation status. Bird Study, 55, 314–320. 10.1080/00063650809461537

[ece35472-bib-0108] Warren, D. L. , Glor, R. E. , & Turelli, M. (2010). ENMTools: A toolbox for comparative studies of environmental niche models. Ecography, 33, 607–611. 10.1111/j.1600-0587.2009.06142.x

[ece35472-bib-0109] Warren, D. , & Seifert, S. (2011). Environmental niche modeling in Maxent: The importance of model complexity and the performance of model selection criteria. Ecological Applications, 21, 335–342. 21563566 10.1890/10-1171.1

[ece35472-bib-0110] Warren, D. L. , Wright, A. N. , Seifert, S. N. , & Shaffer, H. B. (2014). Incorporating model complexity and spatial sampling bias into ecological niche models of climate change risks face by 90 California vertebrate species of concern. Diversity and Distributions, 20, 334–343.

[ece35472-bib-0111] White, G. C. , & Burnham, K. P. (1999). Program MARK: Survival estimation from populations of marked animals. Bird Study, 46, 120–138. 10.1080/00063659909477239

[ece35472-bib-0112] White, G. C. , & Garrott, R. A. (1990). Analysis of wildlife radio‐tracking data. New York, NY: Academic Press.

[ece35472-bib-0113] Wiens, J. A. (1989). Spatial scaling in ecology. Functional Ecology, 3, 385–397. 10.2307/2389612

[ece35472-bib-0114] Williams, T. D. , & Cooke, F. (1994). Fitness consequences of parental behavior in relation to offspring number in a precocial species: The lesser snow goose. The Auk, 111, 563–572.

[ece35472-bib-0115] Zar, J. H. (1999). Biostatistical Analysis (4th ed.). Upper Saddle River, NJ: Prentice Hall.

[ece35472-bib-0116] Zhao, J.‐M. , Fang, Y. , Lou, Y.‐Q. , Swenson, J. E. , & Sun, Y.‐H. (2018). Brood rearing has an immediate survival cost for female Chinese Grouse *Tetrastes sewerzowi*. 2018. Journal of Ornithology, 159, 1019–1029. 10.1007/s10336-018-1578-4

