# Supplementary material for: Behavioral modifications lead to disparate demographic consequences in two sympatric species
Source: Ecol Evol. 2019 Jul 24;9(16):9273–89. doi: 10.1002/ece3.5472 (PMC6706238; doi:10.1002/ece3.5472)

Appendix 1. Ground cover categories for vegetation classification in 2013-2014 at Beaver River Wildlife Management Area in western Oklahoma, USA.

| Land-cover class | Dominant vegetation |
| --- | --- |
| Mixed shrub | Common shrubs include fragrant sumac (*Rhus aromatica*), sand plum (*Prunus angustifolia*), sand sagebrush (*Artemisia filifolia*), and yucca (*Yucca Glauca*). |
| Sand sagebrush | Areas dominated by ground cover of sand sagebrush. Grasses and forbs common but not dominant throughout. |
| Mixed-grass | Common grasses include switchgrass (*Panicum virgatum*), indian grass (*Sorghastrum nutans*), big bluestem (*Andropogon gerardii*), little bluestem (*Schizachyrium scoparium*), and giant sandreed. Common forbs include sand lily (*Menzelia Spp.*), western ragweed (*Ambrosia psilostachya*), common ragweed (*Ambrosia artemisiifolia*), and sunflower (*Helianthus spp.*). |
| Short-grass/yucca | Common grasses include blue grama (*Bouteloua gracilis*), and buffalo grass (*Bouteloua dactyloides*). Dominant shrub is yucca. |
| Sparse vegetation | Typically consists of exposed bare ground with little vegetation cover. |
| Bare Ground | Exposed soils, roads, parking areas, oil and gas pads |
| Salt Cedar | A riparian area typically dominated by salt cedar (*Tamarix spp.*). Common grasses include sand dropseed (*Sporobolus cryptandrus*), fall witchgrass (*Digitaria cognata*), and sand lovegrass (*Eragrostis trichodes*). |
| Open Water | Surface water, including both natural (river) and artificial (holding ponds) sources. |
| Developed areas | Residential areas, houses, and buildings |
| Agriculture/Food Plot | Areas currently under cultivation in addition to wildlife food plots |

Appendix 2. Principal components analysis (PCA) results for daily weather conditions^a,b^ during the summers of 2013-2014 at Beaver River WMA, Oklahoma, USA. The two principal components (PC1 and PC2) were used as independent variables in northern bobwhite (*Colinus virginianus*) and scaled quail (*Callipepla squamata*) chick survival analyses.


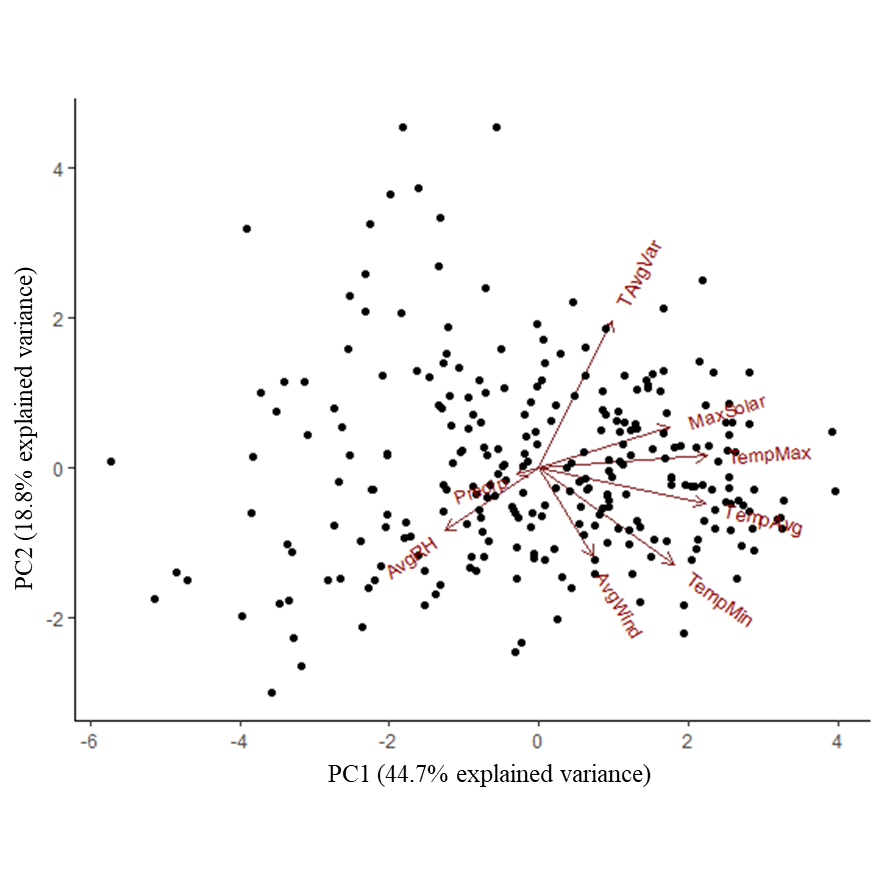


^a^Weather conditions were obtained from three weather stations located on the WMA which collected conditions every hour.

^b^Variables used in PCA analysis: TAvgVar (average daily variance in ambient temperature [°C]), TempMax (maximum daily ambient temperature [°C]), TempAvg (average daily ambient temperature [°C]), TempMin (minimum daily ambient temperature [°C]), MaxSolar (maximum daily solar radiation [W/m^2^]), AvgWind (average daily wind speed [km/h]), Precip (daily precipitation [mm]), and AvgRH (average daily relative humidity [%]).

Appendix 3. Goodness of variance fit values^a^ to determine the optimum number^b^ of data categories for habitat suitability values in the survival analysis of northern bobwhite (*Colinus virginianus*) and scaled quail (*Callipepla squamata*) chicks from 2013-2014 at Beaver River WMA, Oklahoma, USA. Categories were determined using the Jenks natural breaks classification method.

^a^Lines represent: black solid (bobwhite 2 m), black dashed (bobwhite 30 m), grey solid (scaled quail 2 m), grey dashed (scaled quail 30 m).

^b^Number of data categories were determined based on when the goodness of variance fit hit a point of inflection (increased at a decreasing rate).

Appendix 4. Relationship between environmental layers and the probability of habitat suitability at the 2 m grain for northern bobwhite (*Colinus virginianus*) broods during the breeding seasons from 2013-2014 at Beaver River WMA, Oklahoma, USA. Response curves indicate the mean response across 10 replicated Maxent runs and the +/- one standard deviation (grey).

**
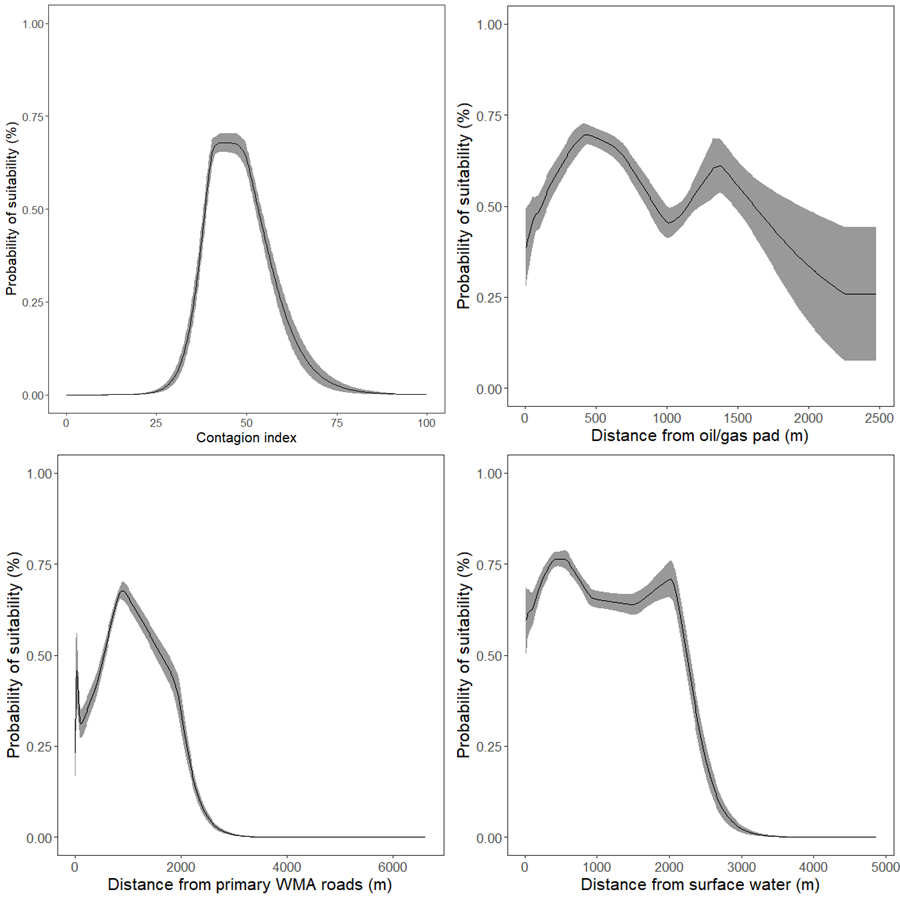
**

Appendix 5. Relationship between environmental layers and the probability of habitat suitability at the 30 m grain for northern bobwhite (*Colinus virginianus*) broods during the breeding seasons from 2013-2014 at Beaver River WMA, Oklahoma, USA. Response curves indicate the mean response across 10 replicated Maxent runs and the +/- one standard deviation (grey).

**
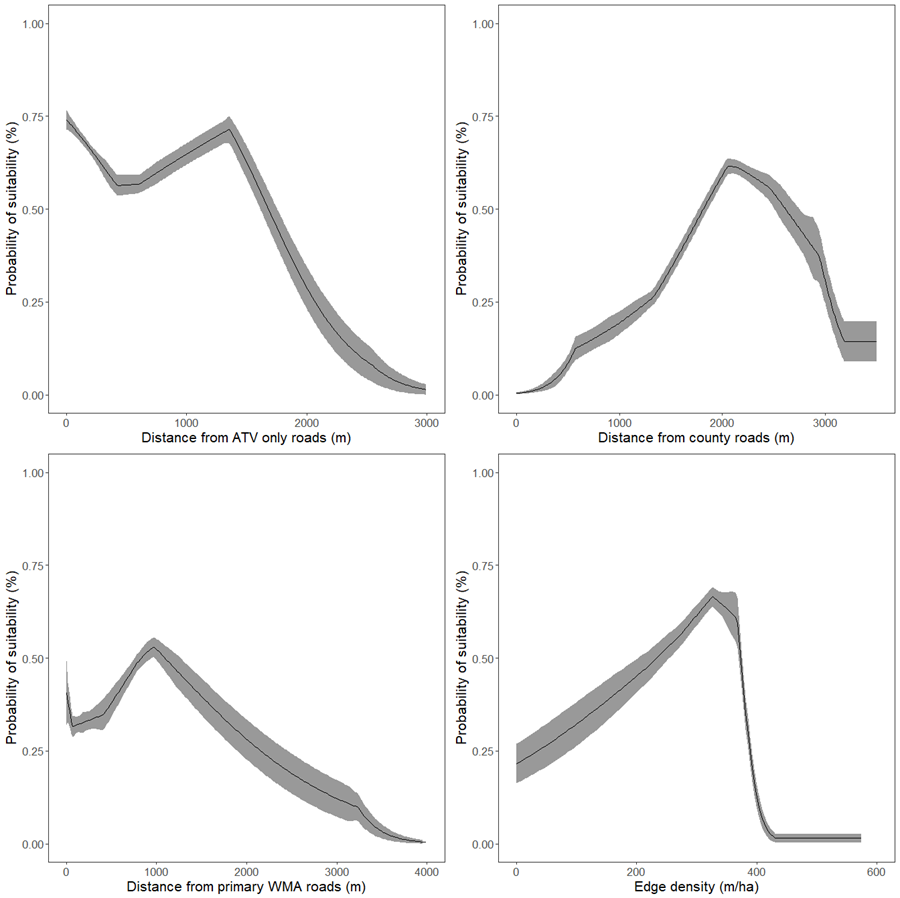
**

Appendix 6. Relationship between environmental layers and the probability of habitat suitability at the 2 m grain for scaled quail (*Callipepla squamata*) broods during the breeding seasons from 2013-2014 at Beaver River WMA, Oklahoma, USA. Response curves indicate the mean response across 10 replicated Maxent runs and the +/- one standard deviation (grey).


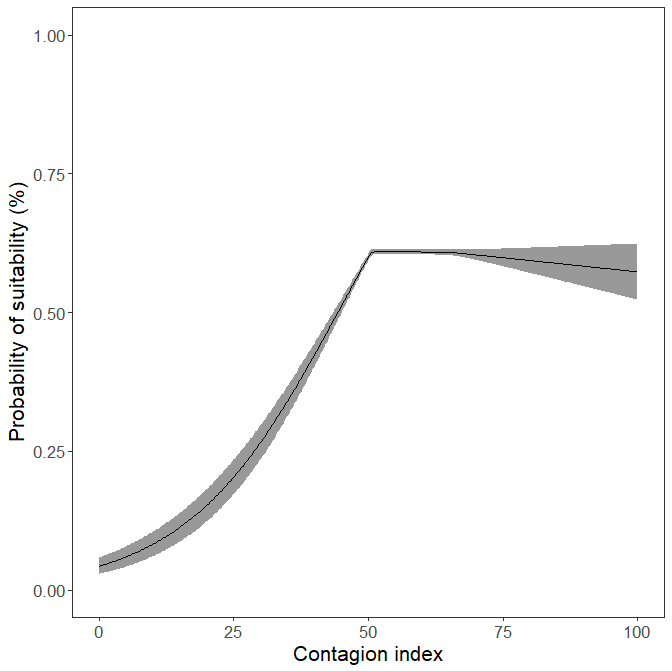

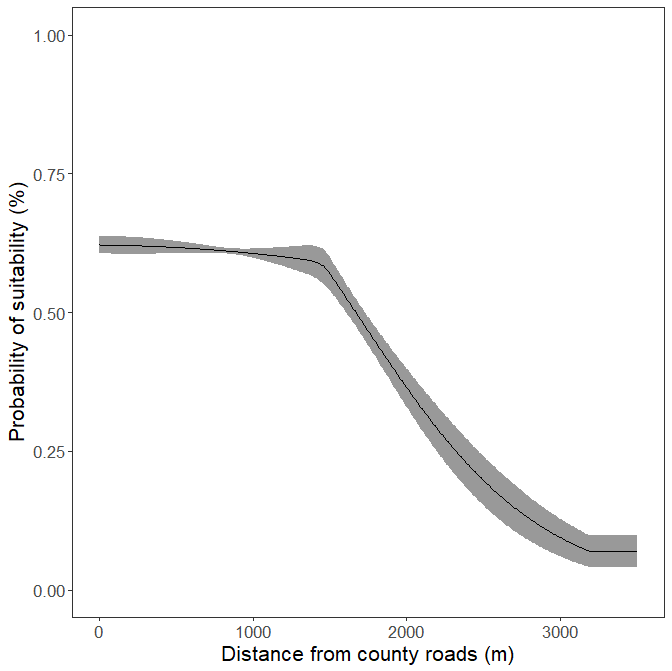

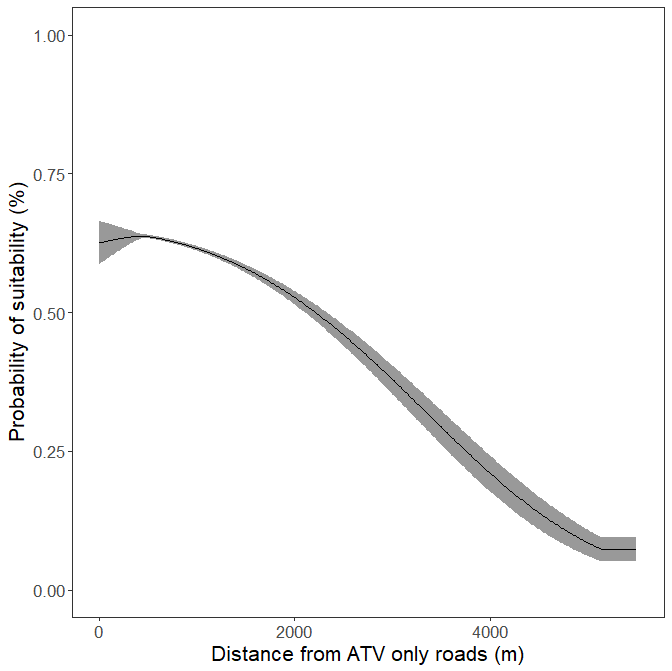

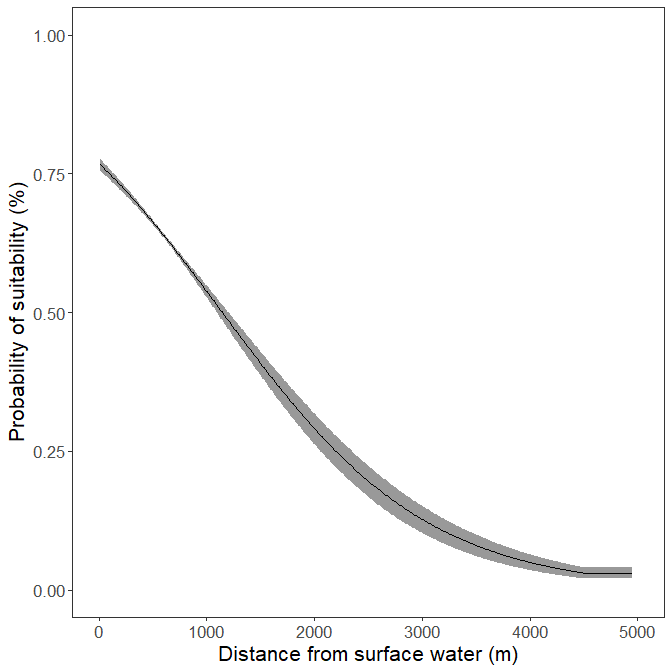

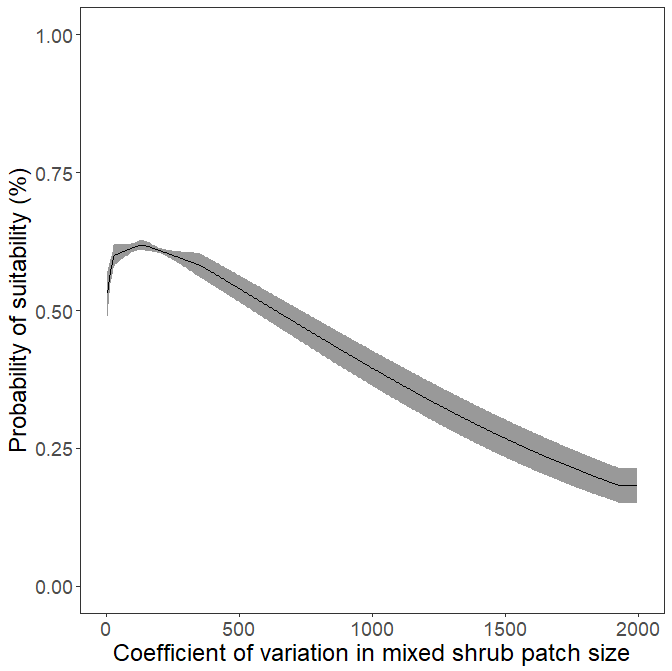


Appendix 7. Relationship between environmental layers and the probability of habitat suitability at the 30 m grain for scaled quail (*Callipepla squamata*) broods during the breeding seasons from 2013-2014 at Beaver River WMA, Oklahoma, USA. Response curves indicate the mean response across 10 replicated Maxent runs and the +/- one standard deviation (grey).


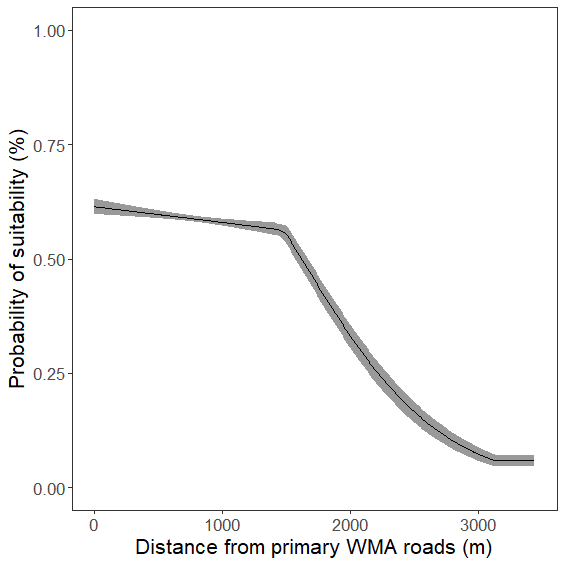

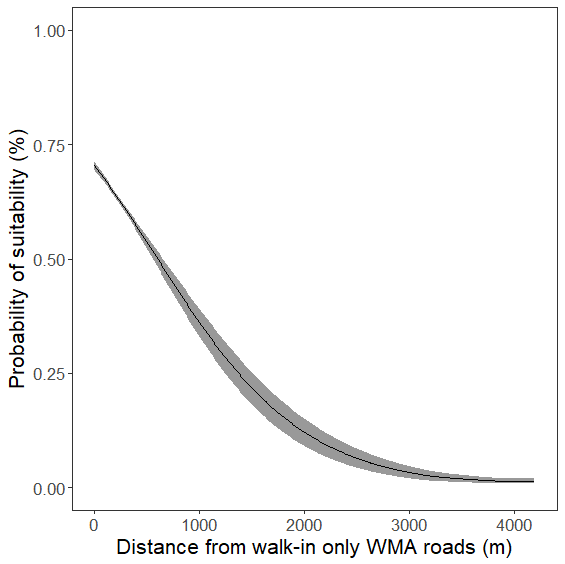

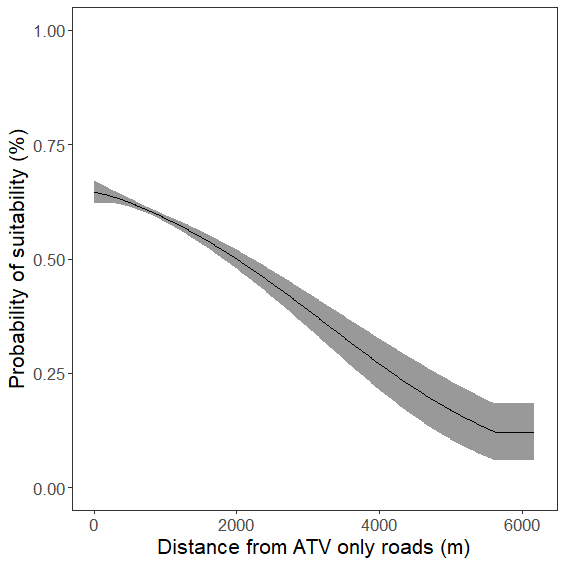

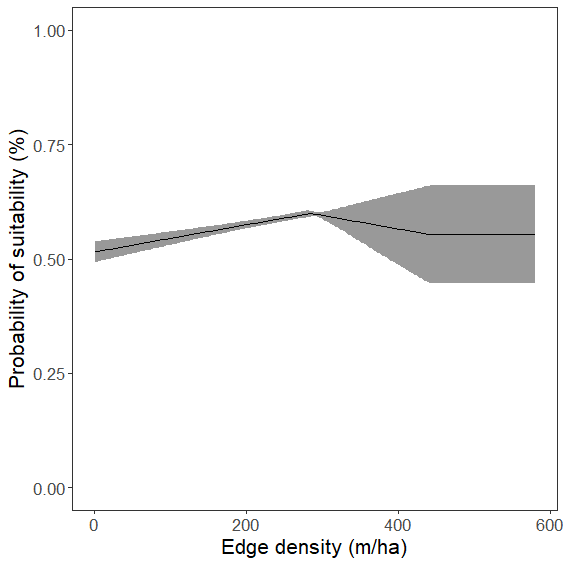

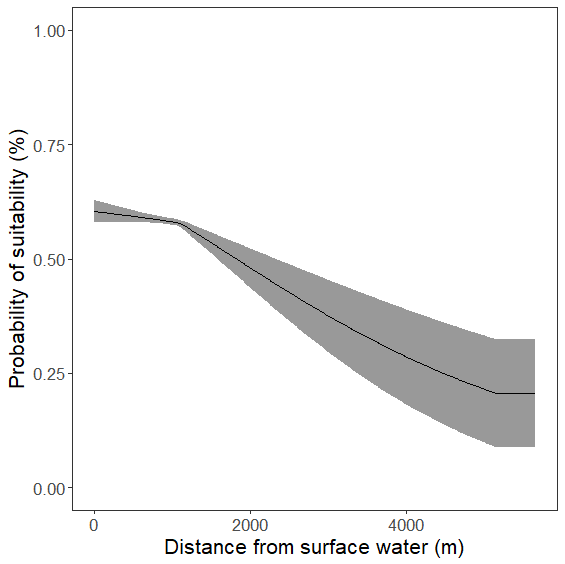

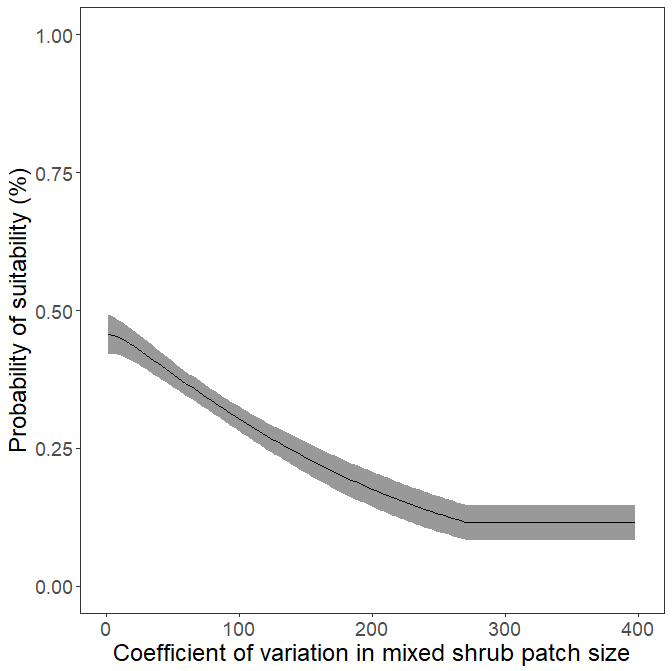


Appendix 8.Variables used in the final first order Maxent models for northern bobwhite (*Colinus virginianus*) and scaled quail (*Callipepla squamata*) non-brooding adults across two grains during the breeding seasons from 2013-2014 at Beaver River WMA, Oklahoma, USA.

| Species | Grain (m) | Variable | Contribution (%) |
| --- | --- | --- | --- |
| Northern bobwhite | 2 | Edge density (m/ha) | 38.3 |
|  |  | Distance from county roads (m) | 36.4 |
|  |  | Distance from walk-in only roads (m) | 25.3 |
|  | 30 | Distance from county roads (m) | 47.6 |
|  |  | Distance from walk-in only roads (m) | 31.9 |
|  |  | Edge density (m/ha) | 15.9 |
|  |  | Distance from surface water (m) | 4.6 |
| Scaled quail | 2 | Distance from walk-in only roads (m) | 58.5 |
|  |  | Edge density (m/ha) | 10.7 |
|  |  | Distance from county roads (m) | 8.3 |
|  |  | Distance from surface water (m) | 7.5 |
|  |  | Coefficient of variation in mixed shrub patch size | 7.4 |
|  |  | Distance from ATV only roads (m) | 5.3 |
|  |  | Distance from primary WMA roads (m) | 2.3 |
|  | 30 | Distance from walk-in only roads (m) | 54.3 |
|  |  | Distance from surface water (m) | 13.3 |
|  |  | Distance from county roads (m) | 13.1 |
|  |  | Coefficient of variation in mixed shrub patch size | 9.8 |
|  |  | Vegetation type | 5.2 |
|  |  | Distance from ATV only roads (m) | 4.3 |

Appendix 9. Relationship between environmental layers and the probability of habitat suitability at the 2 m grain for northern bobwhite (*Colinus virginianus*) non-brooding adults during the breeding seasons from 2013-2014 at Beaver River WMA, Oklahoma, USA. Response curves indicate the mean response across 10 replicated Maxent runs and the +/- one standard deviation (grey).


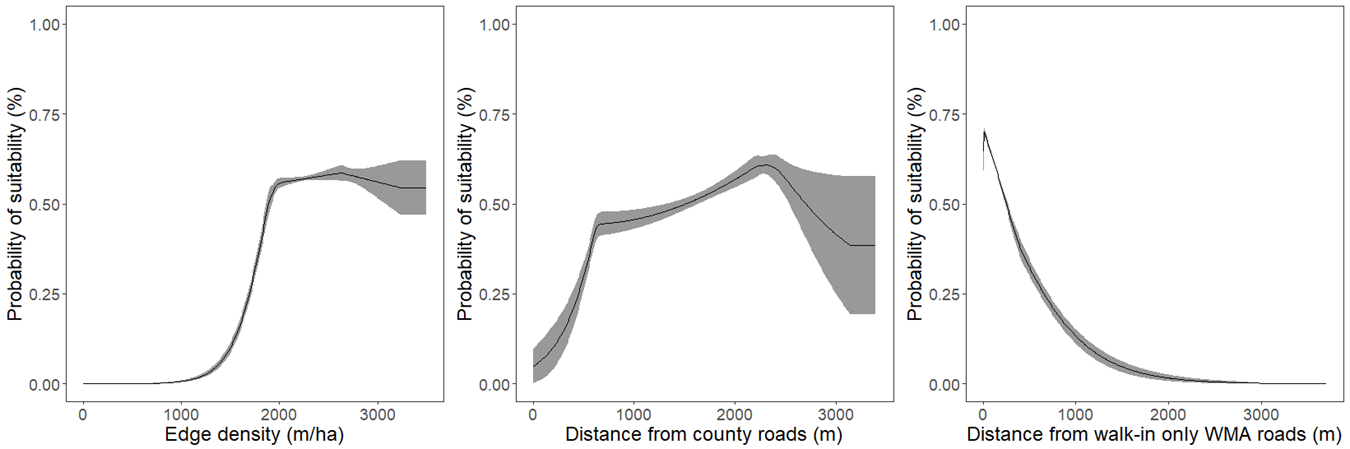


Appendix 10. Relationship between environmental layers and the probability of habitat suitability at the 30 m grain for northern bobwhite (*Colinus virginianus*) non-brooding adults during the breeding seasons from 2013-2014 at Beaver River WMA, Oklahoma, USA. Response curves indicate the mean response across 10 replicated Maxent runs and the +/- one standard deviation (grey).


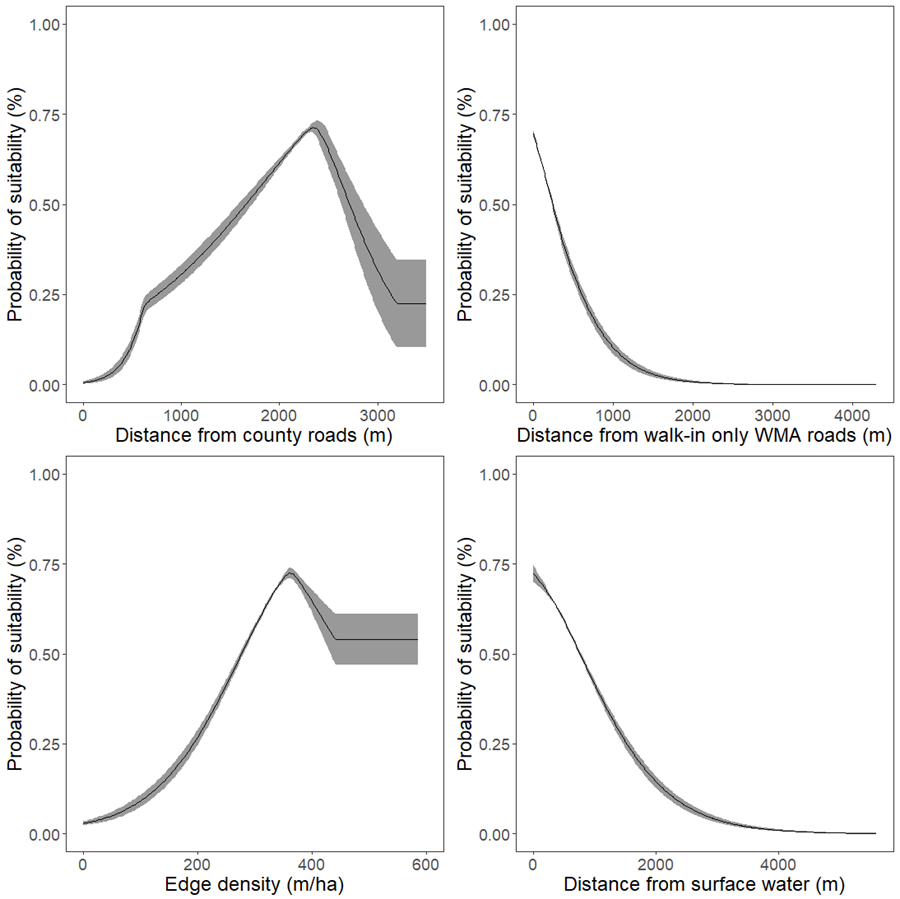


Appendix 11. Relationship between environmental layers and the probability of habitat suitability at the 2 m grain for scaled quail (*Callipepla squamata*) non-brooding adults during the breeding seasons from 2013-2014 at Beaver River WMA, Oklahoma, USA. Response curves indicate the mean response across 10 replicated Maxent runs and the +/- one standard deviation (grey).


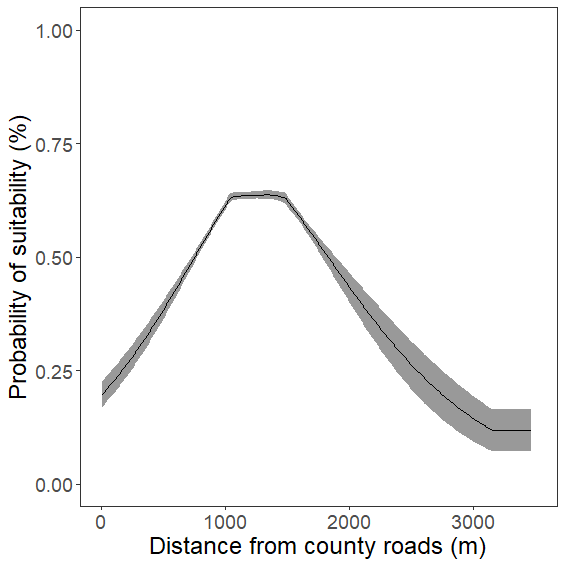

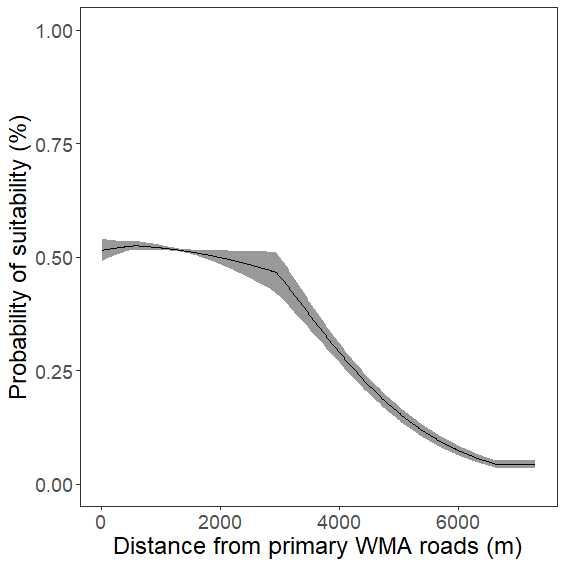

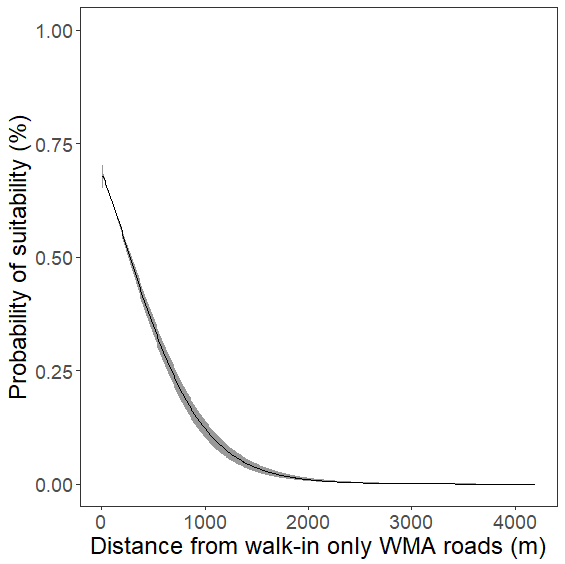

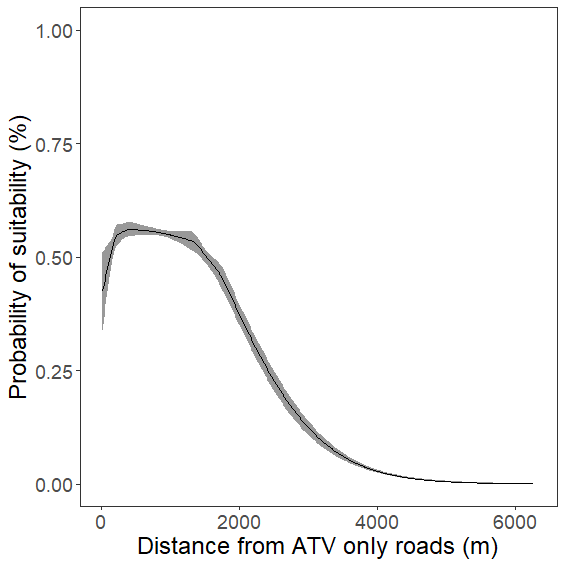

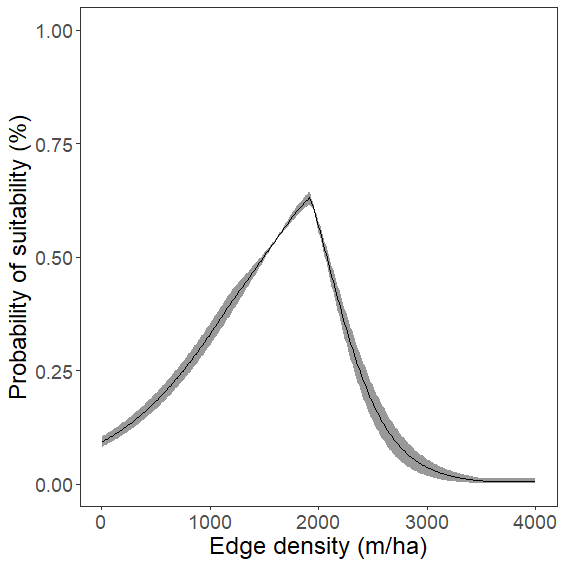

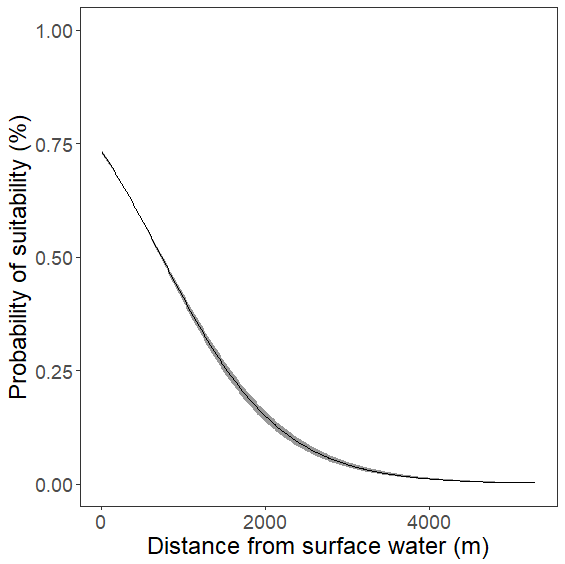

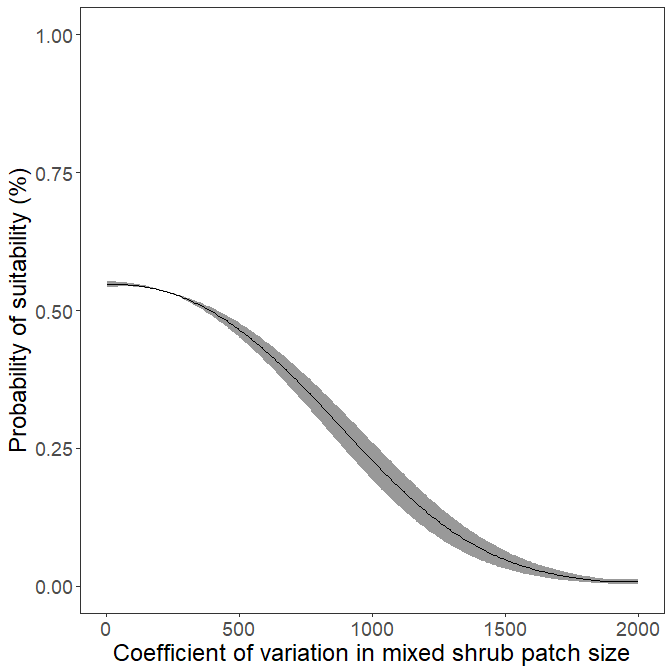


Appendix 12. Relationship between environmental layers and the probability of habitat suitability at the 30 m grain for scaled quail (*Callipepla squamata*) non-brooding adults during the breeding seasons from 2013-2014 at Beaver River WMA, Oklahoma, USA. Response curves indicate the mean response across 10 replicated Maxent runs and the +/- one standard deviation (grey).


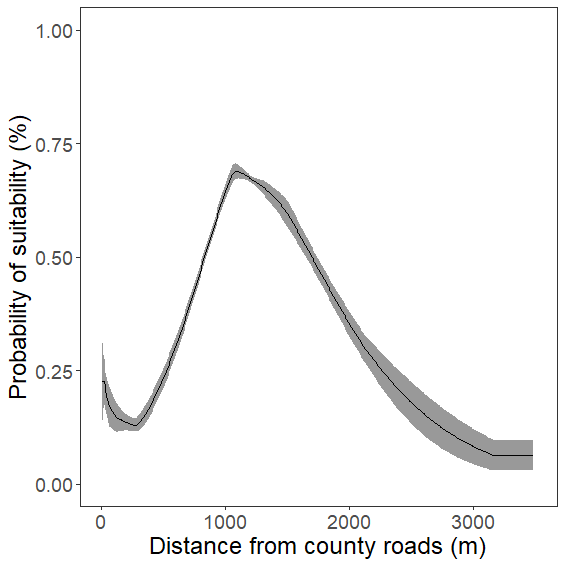

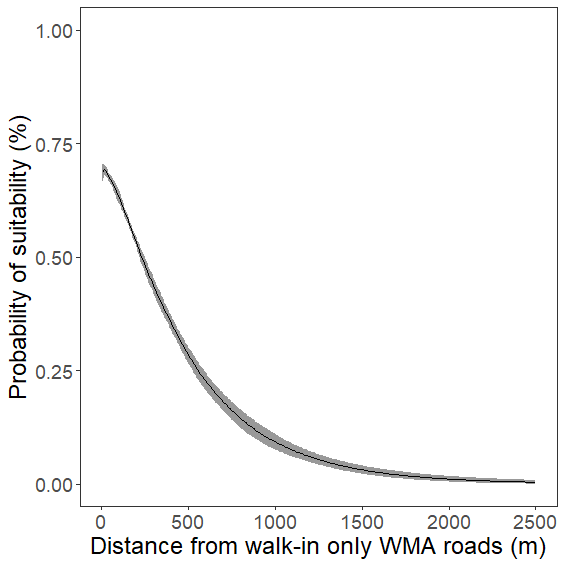

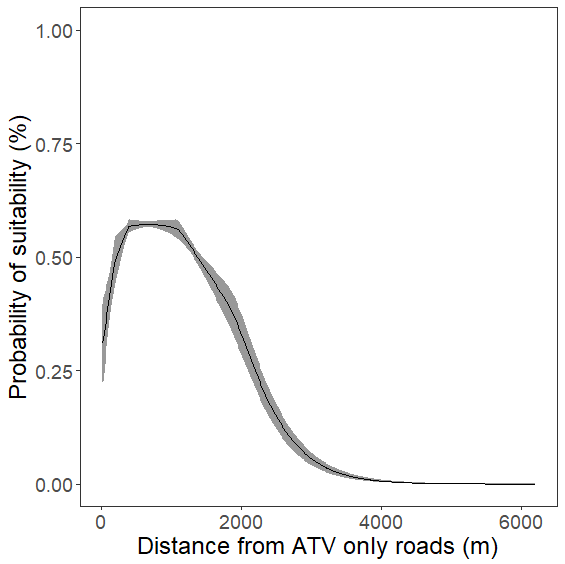

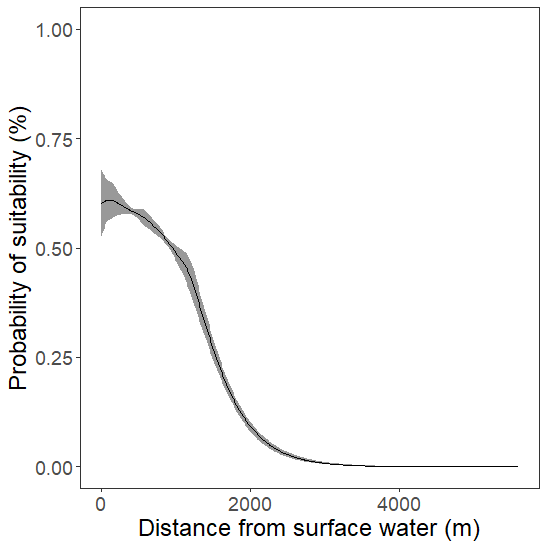

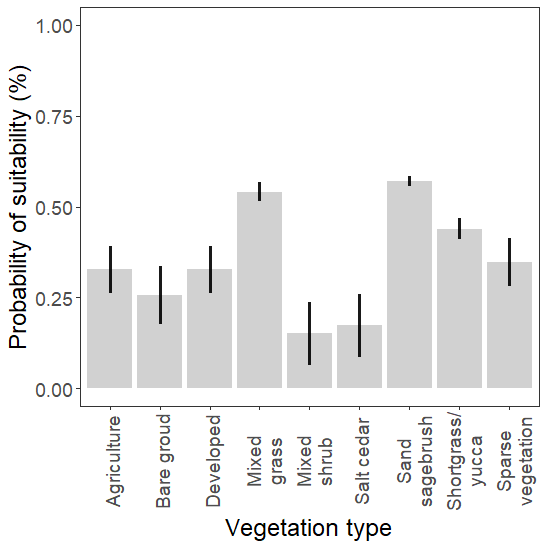

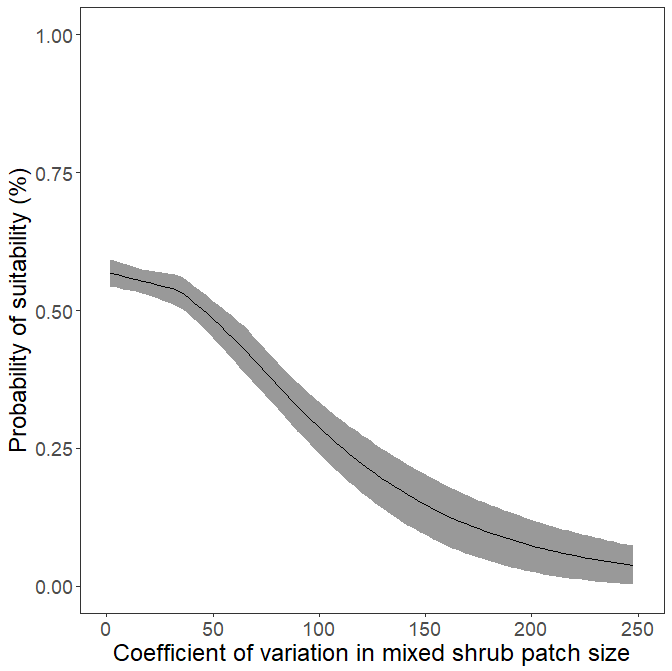

Supplement: Supplementary file 1 [file ECE3-9-9273-s001.docx]
